# Genomic variations and epigenomic landscape of the Medaka Inbred Kiyosu-Karlsruhe (MIKK) panel

**DOI:** 10.1186/s13059-022-02602-4

**Published:** 2022-02-21

**Authors:** Adrien Leger, Ian Brettell, Jack Monahan, Carl Barton, Nadeshda Wolf, Natalja Kusminski, Cathrin Herder, Narendar Aadepu, Clara Becker, Jakob Gierten, Omar T. Hammouda, Eva Hasel, Colin Lischik, Katharina Lust, Natalia Sokolova, Risa Suzuki, Tinatini Tavhelidse, Thomas Thumberger, Erika Tsingos, Philip Watson, Bettina Welz, Kiyoshi Naruse, Felix Loosli, Joachim Wittbrodt, Ewan Birney, Tomas Fitzgerald

**Affiliations:** 1 European Molecular Biology Laboratory, European Bioinformatics Institute, Wellcome Genome Campus, Hinxton, Cambridge, UK; 2 Institute of Biological and Chemical Systems, Biological Information Processing (IBCS-BIP), Karlsruhe Institute of Technology, 76131 Karlsruhe, Germany; 3 Centre for Organismal Studies, University of Heidelberg, Campus Im Neuenheimer Feld, Heidelberg, Germany; 4 National Institute for Basic Biology, Laboratory of Bioresources, Okazaki, Japan

**Keywords:** Inbred panel, Medaka, Genetics, Methylation, Structural variation, Graph genome, Long read sequencing, Pan genome, Nanopore

## Abstract

**Background:**

The teleost medaka (*Oryzias latipes*) is a well-established vertebrate model system, with a long history of genetic research, and multiple high-quality reference genomes available for several inbred strains. Medaka has a high tolerance to inbreeding from the wild, thus allowing one to establish inbred lines from wild founder individuals.

**Results:**

We exploit this feature to create an inbred panel resource: the Medaka Inbred Kiyosu-Karlsruhe (MIKK) panel. This panel of 80 near-isogenic inbred lines contains a large amount of genetic variation inherited from the original wild population. We use Oxford Nanopore Technologies (ONT) long read data to further investigate the genomic and epigenomic landscapes of a subset of the MIKK panel. Nanopore sequencing allows us to identify a large variety of high-quality structural variants, and we present results and methods using a pan-genome graph representation of 12 individual medaka lines. This graph-based reference MIKK panel genome reveals novel differences between the MIKK panel lines and standard linear reference genomes. We find additional MIKK panel-specific genomic content that would be missing from linear reference alignment approaches. We are also able to identify and quantify the presence of repeat elements in each of the lines. Finally, we investigate line-specific CpG methylation and performed differential DNA methylation analysis across these 12 lines.

**Conclusions:**

We present a detailed analysis of the MIKK panel genomes using long and short read sequence technologies, creating a MIKK panel-specific pan genome reference dataset allowing for investigation of novel variation types that would be elusive using standard approaches.

**Supplementary Information:**

The online version contains supplementary material available at 10.1186/s13059-022-02602-4.

## Background

The Japanese medaka fish (*Oryzias latipes*) has a long history as a vertebrate model organism [1, 2]. We took advantage of its unusually high tolerance to inbreeding to establish the Medaka Inbred Kiyosu-Karlsruhe (MIKK) panel: the largest collection of near-isogenic vertebrate lines derived from a single wild population [3]. In the companion article published with this one, we provide a detailed genetic characterisation of the 80 individual MIKK panel lines [4], based on the alignment of Illumina short reads to the closest, fully assembled reference genome—the southern Japanese medaka inbred strain, *HdrR*. Although this allowed us to discover much of the genetic variation in the MIKK panel relative to *HdrR*, the approach inevitably kept certain variants hidden, including larger and more complex structural variation—“dark variation”—that is likely to have functional consequences for each of the lines. Here, we describe how we used Oxford Nanopore Technologies (ONT) long read sequencing to uncover some of this dark variation in 12 of the MIKK panel lines, giving us a more complete assessment of their genomic variation, and paving the way for future studies to elaborate on how structural variants (SVs) affect phenotypes of interest.

The traditional approach for detecting genetic variation is to align reads to a linear reference genome. There are at least three high-quality medaka reference genomes based on inbred strains from different geographical regions in eastern Asia [5, 6]. These include HdrR (southern Japan), HNI (northern Japan), and HSOK (Korea), all of which have been characterised in depth at both phenotypic and genomic levels [7, 8]. Using such linear reference genomes makes it relatively straightforward to determine the functional consequences of genetic variants relative to those references. Although this reference-anchored approach is convenient, it introduces a “reference bias” that can give rise to an under-representation or even incorrect interpretation of genetic variation [9]. Specifically, it makes it difficult to discover complex structural variation, such as large insertions and nested variations.

Variation pangenome graphs offer a compelling alternative approach, allowing for the representation of different classes of SVs using universal semantics [10–13]. The sequencing costs and mapping ambiguity of short reads has so far hindered the widespread adoption of graph genomes. However, recent advances in long-read sequencing technologies [14, 15], and the availability of efficient graph assembly algorithms [11, 16], now make it possible to generate pangenome graphs from multiple draft assemblies at a reasonable cost. These individual assemblies additionally confer the ability to map and quantify different types of repeats [17, 18], which was previously limited when using short-read technology alone. Pangenome reference graph representations and variation is an active area of research with significant progress having been made in methods for creating, interacting with and interpreting these structures [19] across a variety of organisms including humans [10, 11]. Although much progress is being made, this pangenome approach does come with its own limitations, as the graph representation can be challenging to understand and interpret [20]. There are clear advantages offered by moving away from the linear reference [21] and several path-based variant calling methods have already been developed [9, 22]: however, there can still be a barrier to using graph approaches for researchers who are unfamiliar with these structures. Nevertheless, in this study, we demonstrate how these modern assembly generation and aggregation approaches have allowed for a more complete assessment of genomic variation in 12 of the MIKK panel lines, paving the way for additional medaka research studies to use graph genome variation in addition to, or instead of, the traditional reference-anchored approach. This might involve using novel graph variation in genotype-to-phenotype mapping experiments, or further population-based studies comparing other geographically separated medaka strains across Japan and beyond.

Even when applying the traditional reference-anchored approach, using Illumina short-read sequences together with ONT long-read sequences can create a highly accurate representation of large-scale genomic variation. It is clear that SVs impact important traits in humans [23], and it is essential to accurately characterise them to gain a more complete picture of the variation between genomes [24]. Using the combination of long- and short-read sequences takes advantage of their complementary strengths: long reads can span highly repetitive regions, helping to resolve complex SVs, whereas short reads are often of higher quality overall, allowing increased base-calling and mapping accuracy when used to polish the long-read SV calls. Numerous methods for using both technologies in concert have been developed over the years [25], and although there still remain certain challenges associated with SV detection [26], methods that can leverage the combined information from different modern sequencing technologies are likely to provide the highest accuracy [27]. Here, we show how we used ONT long-read information to discover large SVs in 12 MIKK panel lines using the traditional reference-anchored approach, and how polishing the SVs with Illumina short reads substantially improved their mapping accuracy.

Finally, in addition to enabling the construction of pangenome graphs and the discovery of larger SVs, ONT sequencing also allows one to directly detect DNA modifications, such as DNA methylation [28, 29]. We used ONT here to characterise DNA methylation in 12 MIKK panel lines. Altogether, we demonstrate the advantages of using combined short- and long-read technologies, together with both traditional and modern alignment and assembly approaches, in order to more fully characterise large and complex genomic variation. We show several examples of compelling functional consequences, including rearrangements to the exonic structure or the deletion of whole genes. During this work, we provide extensive custom-designed methods and examples of interacting with and extracting meaningful variant-level information from genome graphs [30], both showcasing complex SVs in medaka fish and making our graph assembly available as a resource for the community. We demonstrate some clear advantages of using the graph-based approach and provide new methods for the downstream interpretation of variation. Ultimately, this more complete assessment of the differences between genomes will lead to a more detailed and sophisticated understanding of how genetic variation causes phenotypic differences.

## Results

### Line-specific assemblies and medaka pangenome graph assembly

We selected 12 MIKK panel lines (including 3 pairs of sibling lines) and sequenced brain samples with ONT long read technology to a median of 20x coverage per line, with 37 million reads overall. We multiplexed 4 samples per PromethION flowcell and obtained more than 10 Gb per sample with a mean genome coverage between 13X and 30X as compared with 31X to 39X for illumina sequencing, and the median N50 of the reads was 7411 bp (Additional file 1: Table S1). The analysis consisted of 4 steps: (1) linear draft assembly for each MIKK line using both short and long reads, (2) pangenome graph construction combining known medaka reference genomes and MIKK panel draft assemblies, (3) alignment of ONT reads to the graph, and (4) extraction of complex structural variations.

### Individual MIKK line assemblies

We generated individual assemblies for each line using a hybrid Illumina/ONT strategy. To this end, we first built a scaffold with ONT data using wtdbg2 [31] and then polished the resulting assemblies using the Illumina reads with Pilon [32]. The quality of the draft assemblies was evaluated with Quast [33] against the HdrR reference assembly, BUSCO [34] using the closest available linage (actinopterygii), and a reference free assessment using Merqury [35]. For the BUSCO results using 3640 BUSCOs from 26 genomes, most MIKK panel assemblies had greater than 50% complete BUSCOs with a median of 59% across the 12 assemblies (the “Methods” section). For the reference-free assessment using Merqury, we observe consistent consensus quality (QV) estimates (Additional file 2: Fig. S1A) and reasonable k-mer completeness values with a median completeness of 78% across all 12 assemblies (Additional file 2: Fig. S1B). Although the level of complete BUSCOs in our draft MIKK panel assemblies is lower than needed for reference grade assemblies, we observe similar levels compared to draft assemblies from other teleost species [36] and reasonable k-mer completeness and QV measures from the Merqury reference free assessment (Additional file 3: Table S2). Although we acknowledge that our draft assemblies could be further improved by more sequencing or the addition of further data types such as chromosome conformation capture (Hi-C), they have proved useful in gaining a more complete understanding of genome variation between panel lines and relative to 3 different gold standard reference genomes (*HdrR*, *HNI*, and *HSOK*). However, since these are draft assemblies, we have been strict in our definition of novel variation requiring support from both DNA-seq and RNA-seq in MIKK panel lines.

**Fig. 1:**
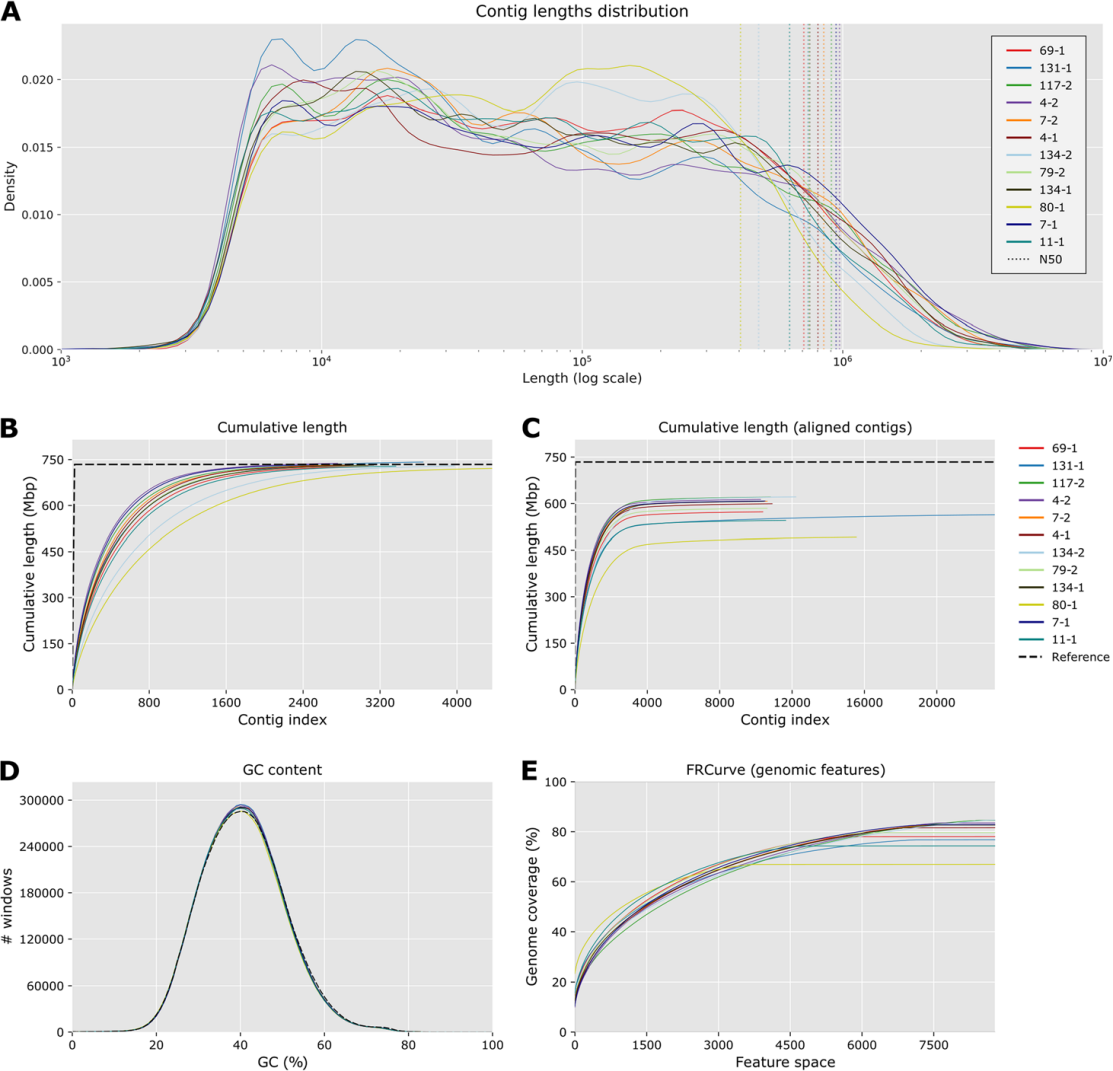
Quality metrics for individual assemblies. **A** Normalised distribution of contigs length for each assembly. Dashed lines represent the N50 values. **B** Cumulative length of contigs. **C** Cumulative length for contig blocks aligned on HdrR, in comparison with the HdrR reference chromosomes (dashed black line). **D** Distribution of CG content of assemblies in comparison with the HdrR reference (dash black line). **E** Feature-response curve for HdrR gene annotation, showing the quality of the assemblies as a function of the maximum number of possible genes allowed in the contigs

**Fig. 2:**
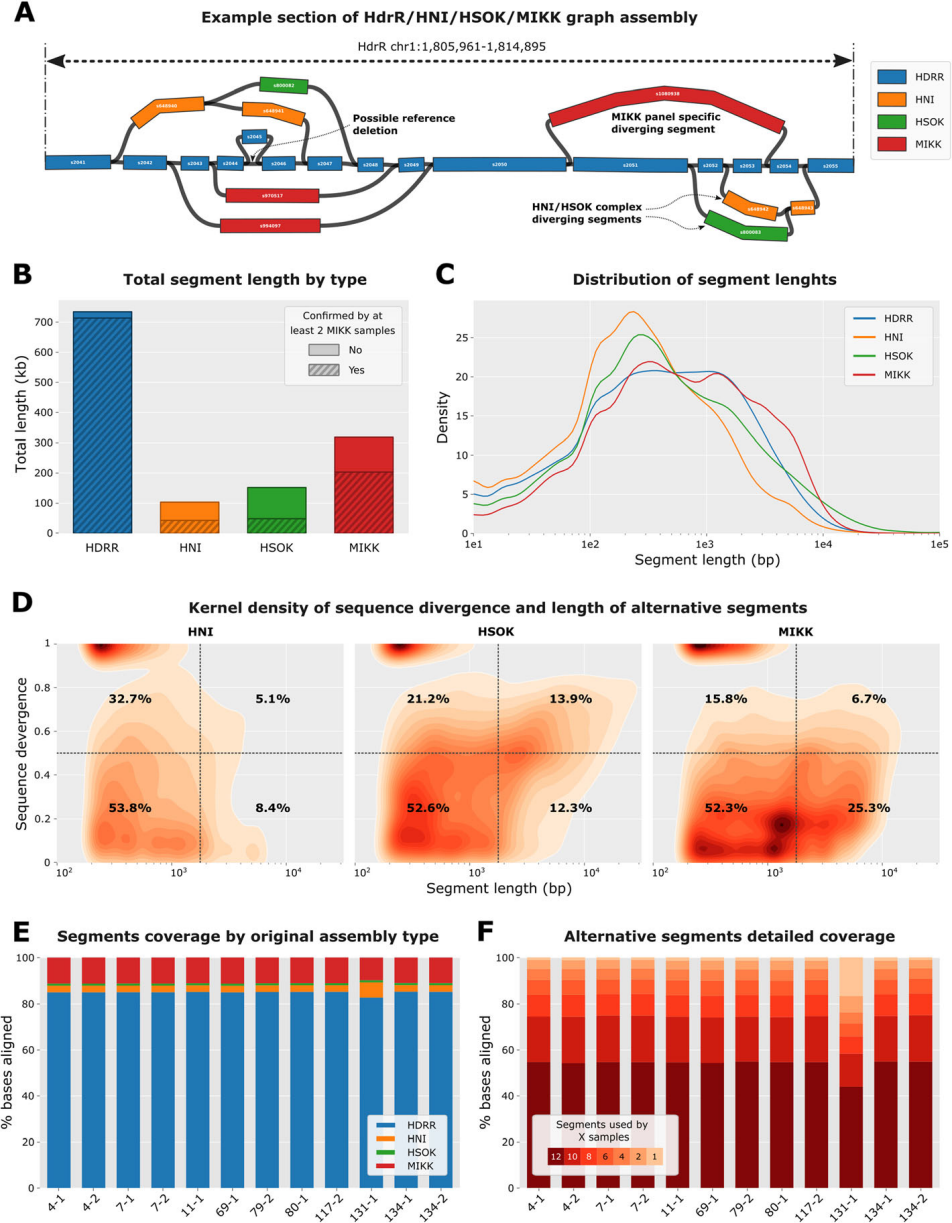
Pangenome graph reference characterisation. **A** Example section of the graph for chromosome 1 showing different paths through the graph via segments originating from the 4 types of assemblies used to build the graph. **B** Total length of segments contained into the graph by type of assembly. Dashed areas represent the proportion of bases for segments covered by at least 2 samples with at least 5% of the average coverage over the HdrR reference segments. **C** Distribution of the length of segment by type of assembly normalised by the total length of segments. **D** Kernel density plots of the length of alternative segments according to their divergence when aligned onto the HdrR reference. The quadrants defined by vertical dashed lines (length = 2kb) and horizontal dashed lines (divergence = 0.5) separate the segments into 4 categories according to their length and divergence score. The numbers displayed correspond to the percentages of segments within each of the 4 quadrants. **E** Percentages of bases from nanopore reads aligning on each type of assembly for the 12 MIKK samples. **F** Detailed percentages of bases aligned on alternative segments for each MIKK sample depending on segments cross-usage by the other samples, from 12 (all other samples) to 1 (only the current sample). A segment was considered used by a sample when its coverage was at least 5% of the average HdrR reference coverage

The assemblies have between 2500 and 4400 contigs amounting to total lengths of 721 to 742 Mb, with N50 values between 404 and 971 kb (Table 1 and Fig. 1A). Assembly lengths are highly consistent with the length of the medaka *HdrR* reference (734 Mb), as are the percentages of CG (Table 1 and Fig. 1D). However, when aligning the contigs to the *HdrR* reference the median alignment length (NA50) scores drop to values between 105 and 280 kb, although many alignments are over 1 Mb long. This is very likely due to the presence of structural variations interrupting alignments and a significant divergence of the MIKK genomes as compared with *HdrR*. Indeed, on average only 80% of the bases from the MIKK panel genomes are aligned unambiguously to the *HdrR* reference and a similar trend is observed for the number of genes covered (Table 1 and Fig. 1C, E). As shown in more details in the following section (Fig. 2B), the majority of the additional 20% present in the MIKK panel occurs in more than one MIKK line. Altogether, this suggests that the MIKK panel line genomes can be reasonably accurately assembled and contain a significant amount of genetic diversity compared with the HdrR line, the closest complete reference assembly.

**Table 1:** Summary statistics of individual MIKK line assemblies

Line id	Number of contigs	GC (%)	Total length	Largest contig	N50	Total aligned length	Largest alignment	NA50
**4-1**	2,886	40.66	730,816,425	5,635,124	802,725	599,170,757	2,340,443	259,311
**4-2**	2,762	40.71	737,637,241	6,376,848	971,613	612,988,975	2,781,976	279,257
**7-1**	2,512	40.69	732,447,291	5,851,261	942,347	608,014,583	2,243,072	265,102
**7-2**	2,892	40.69	732,448,405	4,099,264	845,096	607,409,964	1,761,859	253,015
**11-1**	3,368	40.56	728,542,858	4,525,370	624,727	545,652,612	1,541,261	180,262
**69-1**	3,077	40.59	727,390,278	6,080,511	708,738	573,096,833	1,823,612	220,342
**79-2**	3,053	40.62	730,357,166	6,658,276	742,721	584,535,579	2,384,437	235,007
**80-1**	4,374	40.49	720,948,860	3,238,833	404,886	491,501,885	1,304,457	105,372
**117-2**	2,810	40.73	732,113,747	5,059,334	903,899	620,809,739	2,195,961	270,130
**131-1**	3,651	40.72	741,963,499	5,589,066	737,055	563,763,739	2,231,101	180,572
**134-1**	3,142	40.71	731,842,804	4,138,056	747,064	606,908,341	2,042,573	245,116
**134-2**	3,977	40.75	729,131,624	3,835,876	475,444	621,355,180	1,698,873	209,441

### Pangenome graph assembly and read alignment

To better represent the complexity of the MIKK panel and the relationships with existing medaka reference genomes, we built a pangenome variation graph with minigraph [11] containing all the individual MIKK assemblies together with the *HdrR*, *HNI*, and *HSOK* reference assemblies (http://utgenome.org/medaka_v2). Previous phylogenetic analyses showed that the MIKK panel is genetically closest to *HdrR*, then *HNI* and finally *HSOK* [3]. Thus, we used an iterative strategy to build our pangenome graph, starting with *HdrR* as the primary anchor, followed by *HNI*, then *HSOK* and finally all the MIKK panel assemblies one by one. By doing so, we can identify the segments of the graph that are specific to the MIKK panel, while having information on which is the closest reference for every graph segment. To assess the completeness of graph usage relative to a set of “core genes” in the most closely related gold standard reference (HdrR), we used a similar approach to a previous study [37]. We took all graph segments used by all 12 assemblies (and the HdrR reference) and compared, for each of 24,328 annotated reference genes, the proportion of bases in coding exons that were fully contained within the set of “core graph segments” (the “Methods” section). Next, we defined a gene as being core (“present” in all 12 lines) if over 95% of coding exon bases for that gene were covered. Overall, we found that 78% of annotated reference genes had greater that 95% of their coding exons covered by all 12 lines with only 8.5% of annotated genes missing all coding exons in all lines, leaving an interesting set of 13.5% genes with variable rates of coding sequence fragmentation (Additional file 2: Fig. S2). This suggests that a substantial portion of the annotated coding sequences from the most closely related reference genome (HdrR) may have undergone significant fragmentation and/or deletion in the MIKK panel which is most likely a consequence of complex structural rearrangements.

The presence of non-*HdrR* alternative segments can indicate insertions or significant divergence from the reference, whereas the connection of two non-adjacent *HdrR* segments in linear space is indicative of possible deletions (example shown graphically in Fig. 2A). We obtained a graph containing over 1.1 million segments totaling 1.3 Gb, which is around 1.8 times larger than the *HdrR* reference genome. Together, the 12 MIKK lines bring an additional 211,836 segments to the graph (318 Mb, 24.3% of total) of which 161,533 are covered by at least 2 lines (203 Mb, 20.2%). The segments only found in the MIKK panel have an N50 of nearly 4000 bp and a median sequence identity of 73.7% when aligned to the *HdrR* reference (Table 2 and Fig. 2B–D). In summary, the MIKK panel contains a large number of relatively low divergent paths through the graph mostly consisting of segments ranging from 100 to 5000 bp and cover the majority of annotated *HdrR* reference genes. In comparison both *HNI* and *HSOK* bring fewer segments but with a greater sequence divergence as compared with the *HdrR* reference [38.9% and 60.4% identity, respectively). Interestingly, *HSOK* has a sizable population of long and divergent segments (> 2000 bp and > 0.5 divergence) which represent 13.9% of segments as opposed to 5.1% and 6.7% for *HNI* and MIKK, respectively (Fig. 2D). This is in line with the established phylogeography of Medaka fish, in that the Korean-derived HSOK line is geographically isolated from Japanese medaka lines and earlier branching in evolutionary time.

**Table 2:** Pangenome graph reference statistics. Segment type indicates which assembly the segments originally come from. For the “Segments used by at least 2 MIKK samples” columns, we defined a segment as being used if its coverage is at least 10% of the average coverage over the HdrR reference segments

Segment type	All segments in graph		Segments used by at least 2 MIKK samples		Median segment length	Longest segment	N50	Median % identity
	Length (bp)	# segments	Length (bp)	# segments				
***HdrR***	734,100,826	648,692	713,609,808	615,564	401	675,459	3000	NA
***HNI***	103,507,879	148,689	43,204,187	47,854	239	175,667	2003	62.9%
***HSOK***	152,043,332	100,275	49,055,247	23,881	371	236,527	5803	60.4%
**MIKK**	318,174,656	211,836	203,539,620	161,533	559	89,792	3998	73.7%
**All**	1,307,826,693	1,109,492	1,009,408,862	848,832	389	675,459	3342	NA

Finally, we analysed the graph usage after aligning raw nanopore reads for each individual MIKK sample and computing the coverage for each segment. Overall, the MIKK lines behave similarly in terms of the reference types to which they align, with *HdrR* holding the bulk of the coverage (median = 85.1%) followed by MIKK-specific segments (11%) *HNI* segments (3%) and *HSOK* (0.9%) (Fig. 2E and Additional file 4: Table S3). Among the non-*HdrR* alternative segments, we also investigated the cross-usage of each segment across the 12 MIKK samples. The samples overwhelmingly use segments that are also covered by at least half of all the samples (median = 90.36%) and even by all the samples in the majority of the cases (54.61%) (Fig. 2D and Additional file 5: Table S4). However, there is one notable exception for line 131-1 for which *HNI* type segments get a much larger fraction of the coverage (6.8%). The samples also tend to align on alternative segments supported by fewer samples, with 28.6% of the bases aligned on segments used by fewer than 6 samples, including 16.7% specific to 131-1 line.

### Novel genetic sequences and large-scale insertions and deletions in the MIKK pane

**Fig. 3:**
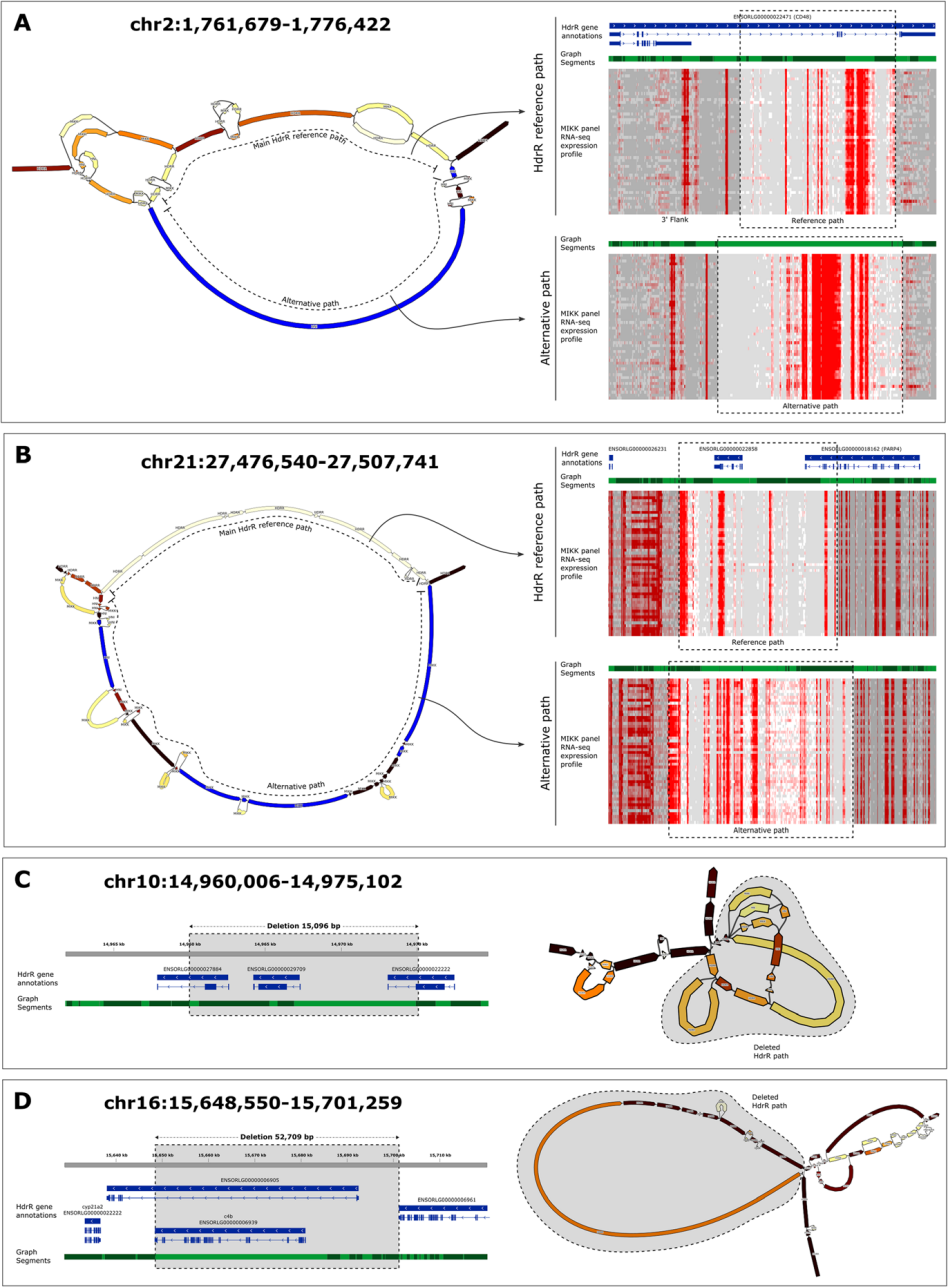
Example structural variations identified in the pangenome graph. **A**, **B** Visualisation of alternative divergent paths in the graph. For both selected examples, the left side panel shows a bandage plot indicating the reference HdrR and the alternative path. Each graph segment is color-coded according to the number of samples with at least 50% of the reference coverage for the ONT DNA-Seq dataset, from white (none) to deep red (all). Blue segments are supported by multiple samples for both Illumina RNA-Seq and ONT DNA-Seq. The right panel shows the linear structure of local assemblies for both the reference and the alternative paths. For the reference, the top blue track represents the existing Medaka HdrR annotations. The light and dark green tracks correspond to the segment layout from the graph. Finally, the heatmaps show the RNA expression intensities for all 50 medaka samples sequenced along the represented sections of the graph (grey = not found, white = less than 5 reads, dark red = more than 100 reads). **C**, **D** Visualisation of large-scale deletions in the graph. For both selected examples, the left panel shows the Medaka HdrR annotations (blue) and the graph segment layout (light and dark green), overlaid with the deletion position (grey rectangle). The bandage plots on the right are color-coded as previously described. The shaded area indicates the reference sequence deletions robustly supported by a direct connection between distant reference segments (link coverage > 50% of reference coverage for at least 9 samples)

Pangenome variation graphs offer new options to discover structural variations that are not available with conventional SV approaches based on linear reference genomes. In particular, they are better suited to represent genomic intervals which accumulated a large number of small variations as divergent alternative paths. We analysed the presence of such paths in our medaka pangenome graph and their potential functional impact. To do so, we identified branches of the graph containing segments which have (1) a low identity compared with the HdrR reference, (2) a robust DNA-Seq and RNA-Seq support from multiple MIKK panel samples, (3) a total cumulative length exceeding 10 kb, and (4) with at least one annotated exon overlapped (see precise criteria in the “Methods”section). With this strict set of criteria, we found 19 such alternative paths in our graph (Additional file 6: Table S5). The 2 examples presented in Fig. 3A/B show the layout of the graph with the reference and the alternative divergent paths. To investigate the precise RNA usage pattern, we generated local linear assemblies for the 2 branches of each selected loci and aligned short RNA-Seq data obtained for 50 MIKK liver samples. In both cases, the exonic coverage pattern over the reference and alternative paths is strikingly different, showing the impact on the transcriptional landscape around these loci of the structural variation.

Large scale rearrangements, deletions in particular, can easily be detected in a graph by analysing the usage of links between segments. We selected links [1] connecting 2 *HdrR* segments distant by more than 10kb, [2] with a strong coverage in multiple MIKK panel samples, and [3] skipping at least 1 annotated exon (see precise criteria in the “Methods” section). We obtained a list of 16 of these large-scale deletions (Additional file 7: Table S6), 2 examples of which are presented in Fig. 3C/D. When lowering the length to 1 kb and not restricting to deletions overlapping exons, we found 2059 such events, showing the widespread occurrence of such deletions with a disruptive potential.

Altogether, our graph genome analysis generated a comprehensive dataset from these 12 lines that has allowed us to identify complex variants. We were able to highlight potential functional consequences including disruption of gene exonic pattern and removal of entire genes. Further computational tools in read mapping and annotation will be needed to robustly identify non-reference genic content in the graph genome. This new way to look at population genomics allowed us to visualize highly complex SVs in medaka fish at an unprecedented resolution and to provide our graph assembly as a resource for the community.

### Structural variation and breakpoint mapping in the MIKK panel

As an alternative to the variation pangenome approach, we also explored structural variation (SVs) in a reference-anchored manner, similar to many human studies [38]. Differences in SVs between panel lines is another important class of genetic variation that could cause or contribute to significant phenotypic differences. Here, we used Nanopore sequencing data obtained for 9 of the 12 selected lines allowing us to characterise larger SVs in the MIKK panel and to create a more extensive picture of genomic rearrangements compared to available medaka reference genomes. We first called structural variants using only the ONT long reads, producing a set of structural variants classified into five types: deletions (DEL), insertions (INS), translocations (TRA), duplications (DUP), and inversions (INV). We then “polished” the called DEL and INS variants with Illumina short reads to improve their accuracy. The polishing process filtered out 7.4% of DEL and 12.8% of INS variants, and adjusted the breakpoints (i.e. start and end positions) for 75–77% of DEL and INS variants in each sample by a mean of 23 bp for the start position, and 33 bp for the end position (Additional file 2: Fig. S3). This process produced a total of 143,326 filtered SVs.

**Fig. 4:**
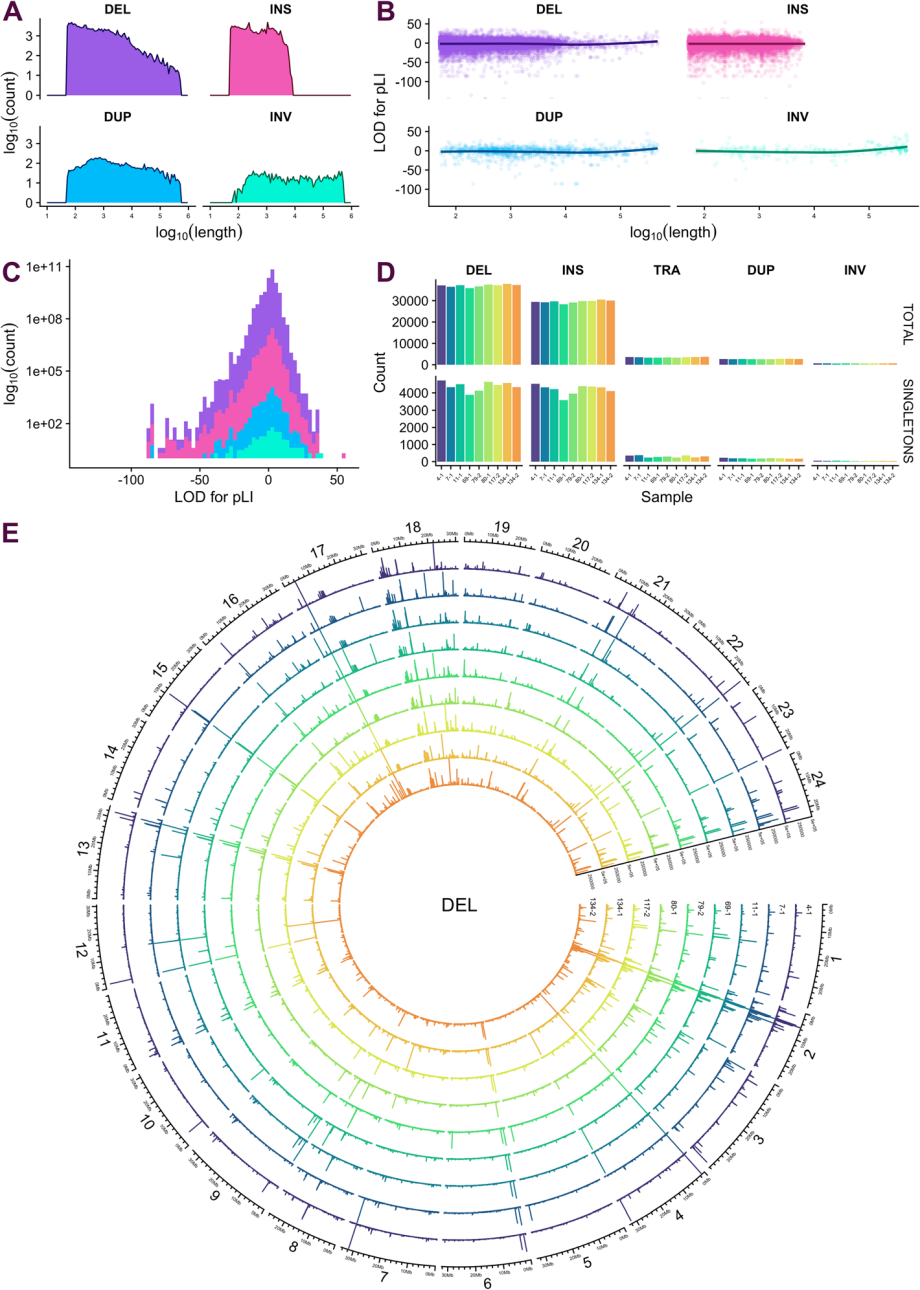
Polished SVs in 9 MIKK panel lines sequenced with ONT. DEL deletion, INS insertion, TRA translocation, DUP duplication, INV inversion. **A** Aggregate log_10_ counts and lengths of distinct SVs by type, excluding TRA. **B** pLI LOD scores in distinct SVs by SV type. **C** Histogram of LOD scores by SV type. **D** Total and singleton counts of SV types per sample. **E** Circos plot showing per-sample distribution and lengths of DEL variants across the genome. Circos figures for each of the other SV types are included in Additional file 8: Fig. S6

The 9 “polished” samples contained a mean per-sample count of approximately 37K DEL variants (12% singletons), 29.5K INS variants (14%), 3.5K TRA variants (9%), 2.5K DUP (7%), and 600 INV (7%) (Fig. 4D). DEL variants were up to 494 kb in length, with 90% of unique DEL variants shorter than 3.8 kb. INS variants were only up to 13.8 kb in length, with 90% of unique INS variants shorter than 2 kb. DUP and INV variants tended to be longer, with a mean length of 19 and 70.5 kb respectively (Fig. 4A). Figure 4E shows the per-sample distribution of DEL variants across the genome. Most large DEL variants over 250 kb in length were common among the MIKK panel lines. A number of large DEL variants appear to have accumulated within the 0–10 Mb region of chromosome 2, which is enriched for repeats in the *HdrR* reference genome (Additional file 2: Fig. S4A)

SVs were generally enriched in regions covered by repeats. While only 16% of bases in the *HdrR* reference were classified as repeats (irrespective of strand), those bases overlapped with 72% of DEL, 63% of DUP, 81% of INV, and 35% of TRA variant regions. However, repeat bases only overlapped with 21% of INS variants. We also assessed each SV’s probability of being loss-of-function (pLI) [39] by calculating the logarithm of odds (LOD) for the pLI scores of all genes overlapping the variant (Fig. 4B, C). 30,357 out of 134,088 DEL, INS, DUP, and INV variants overlapped at least one gene, and 9% of those had a score greater than 10, indicating a high probability that the SV would cause a loss of function. Two INS variants on chr2 had an outlying LOD score of 57 as a result of overlapping medaka gene ENSORLG00000003411, which has a pLI score of 1—the highest intolerance to variants causing a loss of function. This gene is homologous with human genes *SCN1A*, *SCN2A* and *SCN3A*, which encode sodium channels and have been associated with neuronal and sleep disorders. We did not find evidence that longer SVs tended to have a higher probability of causing a loss of function (Fig. 4B).

We compared these polished INS and DEL calls with the high-quality graph-based alternative paths and large-scale deletions, respectively. We found that 2 of the 19 regions covered by graph-based alternative paths (Additional file 6: Table S5), and 4 of the 16 regions covered by graph-based deletions (Additional file 7: Table S6), had no SVs that overlapped those regions at all, which suggests they would have been missed entirely when using a reference-anchored approach alone. With the exception of one alternative path on chromosome 20, the alternative paths were not captured by INS variants, which only covered up to 63% of the bases in each region, and in many cases substantially less. On the other hand, for 8 of the 16 graph-based deletions, the DEL variants covered at least 85% of the bases in those regions. The other 8 graph-based deletions were either not at all covered by DEL variants or only slightly. This indicates that the reference-based approach is better at detecting large-scale deletions than alternative paths (“insertions”), but still misses around half of such variants relative to the graph-based approach.

### Differential methylation analysis

Native DNA sequencing with nanopore technology can be used to robustly detect CpG methylation at single base and single read resolution. There are a number of software methods which can be used to identify methylated positions [28, 29]; however, finding differentially methylated areas of the genome between samples requires processing these methylation calls across samples. To do so, we developed an analytic framework [40] to identify CpG islands of interest in the 12 panel lines sequenced with Nanopore DNA-Seq.

**Fig. 5:**
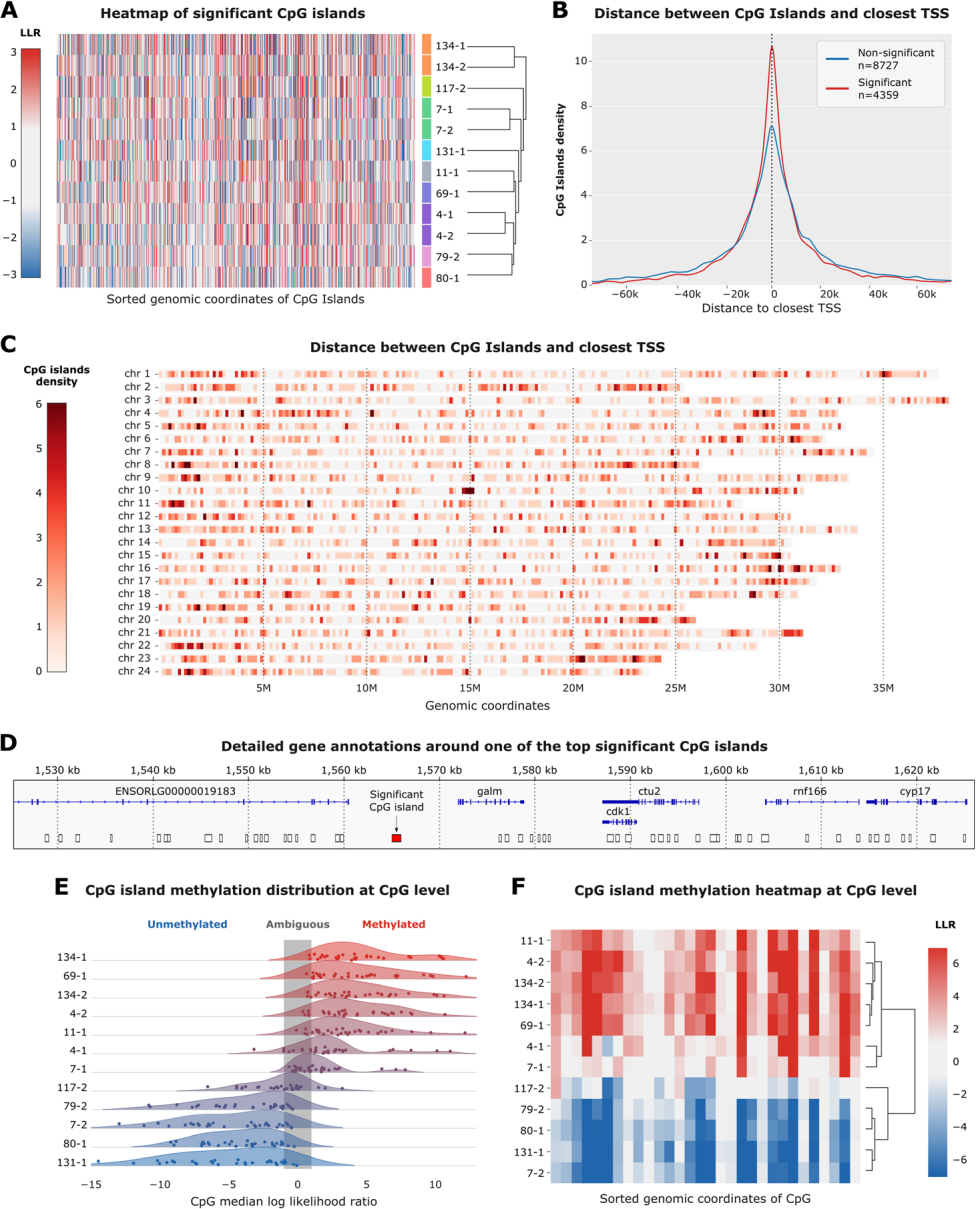
DNA methylation analysis. **A** Heatmap of all significant CpG islands differentially methylated across the MIKK panel samples. CpG islands are sorted by genomic positions and the *X*-axis. Samples are ordered by hierarchical clustering on the *Y*-axis and color coded so that sibling lines are indicated with the same colors. The color scheme is according to the value of the median log-likelihood ratio from −3 (blue) to 3 (red) with ambiguous values between −1 and 1 in white. **B** Distribution of distances to the closest gene TSS for significantly differentially methylated CpG in red (*n*=4249) and all non-significant islands in blue (*n*=8727). **C** Number of significant CpG islands by genomic windows for the 24 HdrR chromosomes, from 0 (white) to 6 (dark red). **D** Example 100kb region containing a significant CpG island in red (chr15:1565040-1565987) as opposed to non-significant ones in white. **E** CpG level log-likelihood ratio kernel density plot for the CpG island highlighted in panel **D**. Samples are sorted on the *Y*-axis by decreasing median llr. Individual CpG values are indicated by dots. **F** Heatmap of log-likelihood ratio with hierarchical clustering by sample for the CpG island highlighted in panel **D**. On the *X*-axis are individual CpGs sorted by genomic position

We were able to use the sequencing data previously generated to explore CpG methylation differences in the brain samples of the 12 selected MIKK panel lines. We found 4459 significantly differentially methylated regions (DMR) among the 271,294 CpG islands included in the analysis (FDR=1%), using a Kruskal-Wallis test. Significant DMR are distributed across the entire genome, with a possible enrichment towards chromosomal extremities (Fig. 5C). In addition, we observed a sharp enrichment of significant DMR near gene transcription start sites compared with non-significant regions (Fig. 5B). When clustering the significant DMR across the entire genome the 3 sib-lines pairs included in the analysis cluster together. This suggests that the methylome is conserved across multiple generations; the simplest explanation being that much of the methylation variation is genetically determined. Detailed interactive reports for the 100 top significant DMR can be found at https://birneylab.github.io/MIKK_genome_companion_paper/DNA_methylation/results/pycometh_html/pycoMeth_summary_report.html [30], and a list of all significant hits is provided as Additional file 9: Table S7. Among the top hits, we found interesting candidates in close proximity to coding genes including *onecut3a*, *gart*, *dnase1*, and *galm.* The *onecut3a* gene is a transcription factor that has been found to have important roles in the development of the liver and pancreas in Zebrafish, in particular biliary development [41]. The *gart* and *galm* genes are enzymes required for *de novo* purine biosynthesis and normal galactose metabolism, respectively, and the *dnase1* gene is an important member of the DNase family involved in actin binding and deoxyribonuclease activity. To investigate differences in expression levels for these four DMRs, we were able to match 8 samples for liver and 6 samples for heart where we also had bulk RNA sequence data available (Additional file 2: Fig. S5). The results here are limited and we expect that in most cases a larger sample size will be required to reliably detect these effects; however, we do observe a significant relationship (*p*=0.002) between methylation likelihood and expression in the liver for the *galm* gene (Additional file 2: Fig. S5D).

## Discussion

Improvements to the accessibility and affordability of long-read genome-sequencing technologies opens up new possibilities for a deeper characterisation of eukaryotic genomes and a more complete understanding of intra-species genetic variation. The standard approach of using a linear reference can only partially handle large and complex structural variation, and a considerable fraction of genetic variation between individuals is masked from sight. Such ‘dark’ genome variation comes in a variety of flavors and scales, including large novel sequence insertions, gene conversion, and introgression from other compatible genomes.

Here, we focused on providing a more complete view of the genetic variation observed across 12 lines from the MIKK panel by assembling a draft genome for each of the lines and then integrating them with three high-quality medaka reference genomes (*HdrR*, *HNI*, and *HSOK*). Each of the 12 MIKK panel lines are inbred lines derived from the same wild founder population from Kiyosu, Japan and can be considered as southern-Japanese strains most closely related to the *HdrR* reference. First, we set out to create high-quality draft genomes for each of the 12 MIKK panel lines using a combination of high-coverage ONT and Illumina sequence data. We observed overall good-quality metrics for the assemblies, with the total sequence lengths being consistently close to the length of the *HdrR* reference genome and similar rates of missing BUSCOs to previous draft assemblies in teleost fish [36]. Interestingly, when aligning these draft assemblies against the *HdrR* reference, NA50 values see a marked decrease, indicating that there is likely to be a considerable fraction of the MIKK panel genomes that is not present in the *HdrR* reference. When aligning these draft assemblies to the *HdrR* reference, we often observed large sequences being split up and fragmented into many smaller sections mapping to different genomic locations, suggesting a marked divergence of the MIKK panel genomes from the *HdrR* reference as a result of many structural variations or novel insertions interrupting the *HdrR* sequence. As more high-quality reference genome assemblies are generated for medaka [42] and other species [43], new approaches for the representation and comparison of genomes are becoming increasingly valuable, as they allow for a deeper understanding of genetic variation within and between species. To further characterise some of the genetic differences within the MIKK panel, and between the MIKK panel and the 3 medaka reference genomes, we used a recently developed graph-based alignment approach, allowing us to represent all 12 MIKK assemblies on the same genome graph together with the 3 reference medaka assemblies. Not only does this approach allow us to represent all MIKK panel assemblies on the same scaffold, but it also provides an intuitive way of assessing how the MIKK panel genomes differ from the genomes of more distant medaka strains.

As the MIKK panel is derived from a southern-Japanese population of wild medaka, the MIKK panel lines are expected to be most closely related to the southern-Japanese *HdrR* reference. This is clearly reflected by the total sequence length within the graph that can be assigned to the *HdrR* reference genome; however, there are an appreciable number of MIKK-panel-specific sequences—the majority of which are supported by 2 or more MIKK panel assemblies. These high-confidence, novel MIKK-panel-specific sequences would be masked when using standard linear-genome-alignment approaches, and would therefore be missing—or incorrectly represented—during downstream analysis, resulting, in the best case, in incomplete genomic variation calls. Furthermore, when looking at novel sequences from the two more distant reference genomes (*HNI* and *HSOK*), it is interesting to observe that the northern-Japanese *HNI* strain contributes fewer novel sequences to the MIKK panel assemblies than the southern-Korean *HSOK* strain, the latter of which is geographically more distant from the MIKK panel’s southern-Japanese founding population. However, for high-confidence sequences (those supported by 2 or more MIKK panel assemblies), the total length of sequences are approximately equal for *HNI* and *HSOK*. The pattern of shared sequence between the MIKK panel lines and the three reference genomes is remarkably consistent across all MIKK panel assemblies with the exception of line 131-1, which shows higher numbers of sequences assigned to *HNI*. This may have been due to the introgression of *HNI* with line 131-1 in the facility during the inbreeding process. Aside from this line, we see little evidence of significant introgression from more distant strains.

The graph-based approach improved our ability to detect large-scale structural variation relative to the traditional reference-anchored approach. Although the process of aligning long reads to the *HdrR* reference and then polishing the calls with short reads revealed a large number of SVs with improved breakpoint-mapping, it still missed around half of the high-quality, large-scale deletions—and most alternative paths—that were discovered through the graph-based approach. This demonstrates the utility of graph genomes to uncover variation that would otherwise be obscured when using the reference-anchored approach. We did observe that the reference-anchored approach had a slight bias in favour of resolving the large graph-based deletions over the large graph-based alternative paths. We also observed a sharp drop in the ability to detect insertions above approximately 10 kb, and given the median long-read length is close to three quarters of this limit, we hypothesise that the ability to resolve large insertions is likely to be directly proportional to read length [44].

It is notable that there are clear genetic consequences for some of the structural variants observed, with a conservative set of 74,271 novel contigs that alter gene content and 11,448 that overlap exonic regions. Having a comprehensive set of functional changes on haplotypes, including these complex variations, is critical for understanding the functional impact of variation. This will be of great importance when using the MIKK panel for genetic association studies of phenotypes, where a full catalog of structural variation will facilitate the attribution of a mapped genetic locus to the correct functional gene. As well as creating a more complete representation of genome structure, the detailed characterisation of genomic variation using advanced assembly aggregation approaches provides important information that can be used to further refine our understanding of gene organisation and function. By using a graph-based approach combined with RNA-sequence alignment, we were able to show distinct expression profile patterns between a standard linear view (or the *HdrR* path) compared to MIKK-panel-specific alternatives for expressed genes. This analysis also shows the value in a graph genome approach to understand functional impacts of structural variation; it is hard to represent some of this variation against a single linear genome, and certainly “nested” variation (that is, variation inside of a region with large variation relative to the reference), which is virtually impossible to handle with a reference-anchored approach.

Variation population graphs are clearly more appropriate for the detection of complex genomic variation across most organisms, including medaka fish, and as the field progresses, we should expect to see further improvements into methods for genome assembly, graph creation, manipulation, and variation interpretation [11, 13, 22, 45]. Here, we provide important additional variation using a graph genome representation of 12 draft assemblies from the MIKK panel and have shown that it is not only possible to detect novel variation from genome graphs, but that some of this variation is likely to have a functional consequence. We provide extensive custom-written code that includes methods used to traverse, detect, and interpret variation using a genome graph approach [30], and we have detailed all methods used to obtain a deeper characterisation of genomic variation in MIKK panel genomes. The results and methods we provide here, with which we have only started to explore novel genomic content relative to three high-quality reference genomes, will be of direct interest to the community and should stimulate further research across a diverse set of medaka fish populations.

An additional and compelling aspect of DNA sequencing using Oxford Nanopore (ONT) is that along with providing long reads suitable for genome assembly; it is also possible to detect DNA modifications, primarily DNA methylation at single-base resolution. We were able to detect thousands of differentially methylated bases using ONT sequence data across 12 MIKK panel lines, providing a further deep characterisation of the genome variation in medaka fish. Interestingly, methylation patterns appear consistent across multiple generations, with the MIKK panel sibling lines clustering together based on their methylation profiles alone. This suggests that, like in other species [46], methylation is a heritable trait in medaka fish capable of persisting across multiple generations and is likely to impact phenotypic traits.

## Conclusions

With this pilot set of 12 MIKK panel lines, we have demonstrated the feasibility of generating independent assemblies for each of the MIKK panel lines and of interrogating the rich functional differences created by their structural variants. Without these long-read-based assemblies and the subsequent variation population graph, we would have been ignorant of the substantial differences in genomic content between the MIKK panel lines. Although there is still a long way to go to make the pangenome variation graph a robust alternative to the linear genome, there is no doubt that it will play a central role in constructing and characterising the ever-expanding catalogue of individual genomes. In addition, we have shown here that the detection of DNA modifications by ONT works robustly in medaka fish. This already provides a useful resource to explore functional differences, but most importantly gives confidence that complete MIKK panel sequencing by ONT will be useful, both for the identification of structural variation, and to provide a key intermediate molecular readout via methylation status. Thus, we present here a methodology for the downstream genome analysis of pangenome graphs that has the potential to become a more widely used approach for population-based studies.

## Methods

### Fish husbandry and dissection

The MIKK panel lines were established from a wild Medaka (*Oryzias latipes*) population as detailed in our back to back companion paper [4]. Liver and brain samples dissection procedures are also described in detail in the companion paper. In this paper, we selected the following lines: **4-1**, **4-2**, **7-1**, **7-2**, 11-1, 69-1, 79-2, 80-1, 117-2, 131-1, **134-1**, and **134-2**. Line ids starting with the same number (in bold) are sibling lines, derived from the same F1 founder family. The selection was done before full stabilisation of the final MIKK panel lines, leading to the following lines not being present in the official 80 stable MIKK lines: 7-1, 131-1, and 134-2.

### Sample preparation and sequencing

Briefly, RNA extraction from liver samples and DNA extraction from brain samples were performed on a Qiagen automated extraction platform using QIAsymphony RNA and DNA Kits, respectively. Samples were prepared for Illumina DNA-Sequencing using the standard PCR-free Illumina protocol [47] and RNA-Sequencing using the NEBNext Ultra II Directional RNA Library Prep Kit following the manufacturer’s instructions.

For Nanopore DNA-Sequencing, brain DNA samples were prepared with the ligation sequencing kit (SQK-LSK109), multiplexed with the native barcoding expansion kit (EXP-NBD104), and finally loaded in a FLO-PRO002 flow-cell on a PromethION instrument, all following the manufacturer’s instructions (Oxford Nanopore, Oxford, UK). To reduce sequencing costs while targeting a coverage of around 15X, we multiplexed 4 samples per flowcell. The 12 MIKK panel samples were selected based primarily on DNA material availability, but within the selectable sample set (those with sufficient material for ONT sequencing), we also ensured that they covered a good representation of the range of homozygosity levels across the MIKK panel and that we included 3 sibling lines.

### Bioinformatic methods and data

Raw sequencing data can be retrieved from ENA linked to the following project ID set out in the “Availability of data and materials” section.

All the scripts and metadata used for this study are extensively described in the associated github repository available at https://github.com/birneylab/MIKK_genome_companion_paper [30].

### Nanopore data processing

#### Basecalling

After nanopore sequencing, raw nanopore data in FAST5 format was transferred securely from Sanger Institute storage to the EBI high performance compute cluster, where all the analyses were performed. FAST5 files were basecalled and demultiplexed according to the 4 expected barcodes for each run with ONT-Guppy (v4.0.14). See detailed analysis and metadata at https://birneylab.github.io/mikk_genome_companion_paper/nanopore_basecalling/ [30].

#### Alignment and QC

We developed a Snakemake pipeline [48] called pycoSnake [49] to run the entire analysis, including mapping, quality control, differential methylation analysis, and structural variation calling. For this study, we ran pycoSnake v0.1a3 (commit hash 6d248c0fddfedd8f27d59b59f94f63f64d16e9bd) [50], DNA_ONT workflow v0.2. All the tools and environment are version controlled in individual conda environments. Briefly, reference genome and annotations were obtained from ensembl Release 99 (Japanese medaka *HdrR* ASM223467v1, https://www.ensembl.org/Oryzias_latipes/Info/Index). Basecalled reads are merged and filtered using pyBiotools v0.2.0.9 [51], then aligned to the reference using Minimap2 v2.15 [52]. Alignments are filtered to keep only high-quality primary reads using pyBiotools v0.2.0.9 [51], and quality control checks are performed using pycoQC v2.5.0.23 [53]. The detailed parameters used to run each tool as well as the sample QC can be found at https://birneylab.github.io/MIKK_genome_companion_paper/Nanopore_processing/ [30]. We assessed assembly quality using QUAST [33], BUSCO [34], and Merqury [35] and provide an overview of this assessment within the results section; for BUSCO, we used the closest available linage dataset (*actinopterygii*), and for Merqury, we used meryl to find the optimum k-mer size of 19. The full results can be found at https://birneylab.github.io/MIKK_genome_companion_paper/Individual_assemblies/ [30].

#### DNA methylation analysis

The differential methylation analysis was performed as part of the pycoSnake pipeline, after the alignment steps described before. In brief, CpG methylated sites are called at single read level with nanopolish call_methylation v0.11.1 [28]. Methylation log likelihood ratio (LLR) are aggregated at genomic position level, then within CpG islands. Finally, for each CpG island with sufficient coverage the differential methylation analysis is performed using pycoMeth v0.4.25 [40]. Briefly, all median LLR values for each CpG positions within a given CpG island are compared between samples using a Kruskal-Wallis test and all resulting *p* values are adjusted for multiple tests using the Benjamini & Hochberg procedure for controlling the false discovery rate (FDR). We also performed extra analyses to produce the final paper figure in a Jupyter notebook. Additional information on the differential methylation analysis can be found at: https://birneylab.github.io/MIKK_genome_companion_paper/DNA_methylation/index [30].

#### Structural variant calling

Structural variant calling was also performed as part of pycoSnake pipeline. Reads were re-aligned with NGMLR v0.2.7, followed by a first round of SV detection with Sniffles v1.0.12 [54]. Variants were subsequently filtered and merged with SURVIVOR v1.0.7 (https://github.com/fritzsedlazeck/SURVIVOR) [55]. Then, a second round of Sniffles SV calling was done using the merge calls to constrain the detection to the common filtered variants previously collected. Finally, all calls are merged in a single unified VCF file. Additional information on the Structural variant calling analysis can be found at: https://birneylab.github.io/MIKK_genome_companion_paper/Nanopore_SV_analysis/ [30]. To polish the calls with Illumina reads, we used the Illumina reads and VCF described in [4] with SViper v2.0.0 [56] to produce a “polished” set of structural variants for 9 of the 12 MIKK panel samples. (Lines 4-2 and 7-2 failed this processing stage without an error message, so were necessarily excluded from the downstream reference-anchored analysis. We also excluded line 131-1 because it had an unusually high level of *HNI* sequence content (see the “Discussion” section) and could therefore bias the results). We used bcftools v1.9 and Picard v2.25.0 [57, 58] to further process the data, then R version 4.0.4 and a suite of R packages [59–70] to carry out the analysis set out in full at: https://birneylab.github.io/MIKK_genome_main_paper/06_Structural_variation.html [30].

#### Prediction and annotation of repetitive and transposable elements

The *RepeatModeler* pipeline (v2.0.0) [71] for the automated de novo identification of repetitive and transposable elements was run on all chromosomes in the *HdrR* genome assembly [8]. RepeatModeler was run with its default parameters and the additional long terminal repeat (LTR) structural discovery sub-pipeline that includes the *LTRharvest* [72] and *LTR_retriever* [73] tools.

The RepeatModeler library of repeats was filtered to remove non-TE protein coding sequences by using a protein BLAST (Altschul et al., 1990) to align (*E* value ≤ 1e-5) the *Oryzias latipes* proteome (Ensembl v99) and *pfam* peptide database (v32) against the RepeatMasker peptide library. Finally, a nucleotide BLAST was used to remove any RepeatModeler repeats that aligned (*E* value ≤ 1e-10) against the corresponding transcripts.

RepeatMasker (v4.1.0) [74] was used to align the chromosomes in the *HdrR* assembly against the filtered RepeatModeler library of consensus repeats and the existing RepeatMasker repeat families.

Additionally, *Exonerate* (Slater and Birney, 2005) was used to align the two subtypes of the *Teratorn* mobile element found in the *Oryzias latipes* genome against the *HdrR* reference. (The *Teratorn* element being the result of a fusion between a *piggyBac* DNA transposon and a member of the *Alloherpesviridae* family [17]).

### Assembly and graph analysis

#### De novo assembly of MIKK panel genomes

For each line, Nanopore FASTQ raw sequences were assembled using the long-read assembler wtdbg2 in Nanopore (ONT) mode to create draft assemblies for the 12 MIKK panel genomes [31]. We then polished each of the draft assemblies with their corresponding ~30X Illumina sequences using 2 rounds of the Pilon [32]. The draft assembly qualities were evaluated using QUAST v5.1.0rc1 [33], and FASTA were deposited at ENA under the same study accession as the nanopore reads (PRJEB43089) [78]. Additional information on the analysis and access to raw data can be obtained at https://birneylab.github.io/MIKK_genome_companion_paper/Individual_assemblies/ [30].

#### Variation pangenome graph assembly

On top of the MIKK panel line draft assemblies, we also used 3 high-quality medaka reference assemblies *HdrR*, *HNI*, and *HSOK*, including unanchored contigs, to scaffold the graph (http://utgenome.org/medaka_v2). Prior pangenome assembly each contig from every reference was prefixed with the reference name it belongs to, to allow unambiguous identification of the origin of graph segments (eg, HdrR_1 for chromosome 1 of the *HdrR* reference). We assembled the graph pangenome using minigraph2 v0.10 [11] (-x ggs mode) adding iteratively each reference in the following order HdrR, *HNI*, and *HSOK*, then the MIKK lines 69-1, 131-1, 117-2, 4-2, 7-2, 4-1, 134-2, 79-2, 134-1, 80-1, 7-1, and finally 11-1. The resulting graph in rGFA format was parsed to extract descriptive statistics as well as graph anchored annotations for Bandage [75] and IGV [76] using python scripting. The analysis notebook and the raw data can be found at https://birneylab.github.io/MIKK_genome_companion_paper/Graph_assembly/ [30].

#### Graph alignment and segment usage analysis

We aligned the DNA-Seq nanopore reads for each of our 12 MIKK samples to the pangenome graph using minigraph2 v0.10 [11] (-x lr mode) and obtained alignment files in GAF format. We also aligned the 50 Illumina RNA-Seq datasets obtained from MIKK line liver samples described in [4] to the graph. However, since pair-end mapping is not supported by minigraph, we first merged overlapping pairs together using Flash v1.2.11 [77], then aligned the merged reads to the graph with minigraph2 v0.10 [11] (-x sr mode). We then computed the length normalised coverage of segments and junctions between segments for each sample and generated statistics on graph segment usage per samples, using python scripting. The analysis notebook and the raw data can be found at https://birneylab.github.io/MIKK_genome_companion_paper/Graph_usage/ [30].

#### Core gene analysis and the definition of gene presence/absence

We used the read alignment and graph segment usage profiles from above to define “core graph segments” as those segments from the graph that were used by all MIKK panel lines (and the HdrR reference). For the assessment of core gene presence, we calculated the proportion of exonic bases that were covered by these core graph segments for 24,328 annotated reference genes. A gene was considered core if over 95% of its exonic bases were present in segments used by all MIKK panel lines and variable if it was absent in at least one line.

#### Graph structural variation analysis

Based on the normalised coverage of graph segments and junctions, we investigated the presence of 2 types of genetic variations in our MIKK panel: large scale divergent insertions with DNA and RNA-Seq supports and complex deletions. For the divergent insertions analysis, we searched for alternative non-*HdrR* paths longer than 10kb, containing segments with a sequence diverging by more than 50%, supported by at least 2/12 samples for DNA-Seq (50% of mean *HdrR* coverage) and 8/50 samples for RNA-Seq (10% of mean *HdrR* coverage) and overlapping at least 1 annotated gene exon. With this very strict set of criteria, we found a set of 19 such paths (Table SX). For the complex deletions, we leveraged the coverage information for the junction/link between segments instead. We identified 16 deletions, supported by junctions connecting 2 *HdrR* segments distant by more than 10kb, 2) with a coverage greater than 50% of the average *HdrR* supported by at least half of the panel lines3) skipping at least 1 full annotated *HdrR* exon. These candidate insertions and deletions were then manually investigated using Bandage [75] for visualisation in graph space and IGV [76] for HdrR anchored linear genome visualisation. The jupyter notebook containing the full analysis and raw data can be found at https://birneylab.github.io/MIKK_genome_companion_paper/Graph_SV/ [30].

## Supplementary Information

**Additional file 1: Table S1.** *Nanopore sequencing metrics.* Metrics from ONT PromethION sequencing.

**Additional file 2: Figures S1-S5.** *Supplementary figures.* Various supplementary figures.

**Additional file 3: Table S2.** *Individual assembly metrics.* Metrics from individual assemblies including BUSCO, k-mer completeness, and QV measures.

**Additional file 4: Table S3.** *Graph segment alignments to reference genomes.* Percentage of graph segments aligned to HdrR, HNI, and HSOK reference genomes. and shared across 12 MIKK panel lines.

**Additional file 5: Table S4.** *Graph segments shared across MIKK panel lines.* Percentage of graph segments shared between the 12 MIKK panel lines.

**Additional file 6: Table S5.** *High-quality graph alternative paths.* Genome coordinates for 19 MIKK panel-specific high-quality graph alternative paths.

**Additional file 7: Table S6.** *High-quality graph deletions.* Genome coordinates for 16 MIKK panel-specific high quality graph deletions.

**Additional file 8: Figure S6.** *Circos plots.* Circos plots for structural variants (DEL, INS, DUP, INV, TRA) discovered by the reference-anchored approach.

**Additional file 9: Table S7.** *Differentially-methylated regions.* Genome coordinates of all significant differentially-methylated regions in 12 MIKK panel lines.

**Additional file 10.** Review history.

## Data Availability

The datasets supporting the conclusions of this article are available in the European Nucleotide Archive (ENA) hosted at the EBI: https://www.ebi.ac.uk/ena/browser/home. The individual raw sequencing datasets are linked to the following project IDs: Nanopore DNA sequencing data: https://www.ebi.ac.uk/ena/browser/view/PRJEB43089 [78] Illumina DNA sequencing data: https://www.ebi.ac.uk/ena/browser/view/PRJEB17699 [79] Illumina RNA sequencing data: https://www.ebi.ac.uk/ena/browser/view/PRJEB43091 [80] All the scripts and metadata used for this study are extensively described in the associated GitHub repository under MIT License available at https://github.com/birneylab/MIKK_genome_companion_paper [30] and zenodo 10.5281/zenodo.5779555 [81].
