# Supplementary material for: Genomic variations and epigenomic landscape of the Medaka Inbred Kiyosu-Karlsruhe (MIKK) panel
Source: Genome Biol. 2022 Feb 21;23:58. doi: 10.1186/s13059-022-02602-4 (PMC8862245; doi:10.1186/s13059-022-02602-4)
Supplement: Supplementary file 2 — Additional file 2: Figures S1-S5. Supplementary figures. Various supplementary figures. [file 13059_2022_2602_MOESM2_ESM.docx]

**
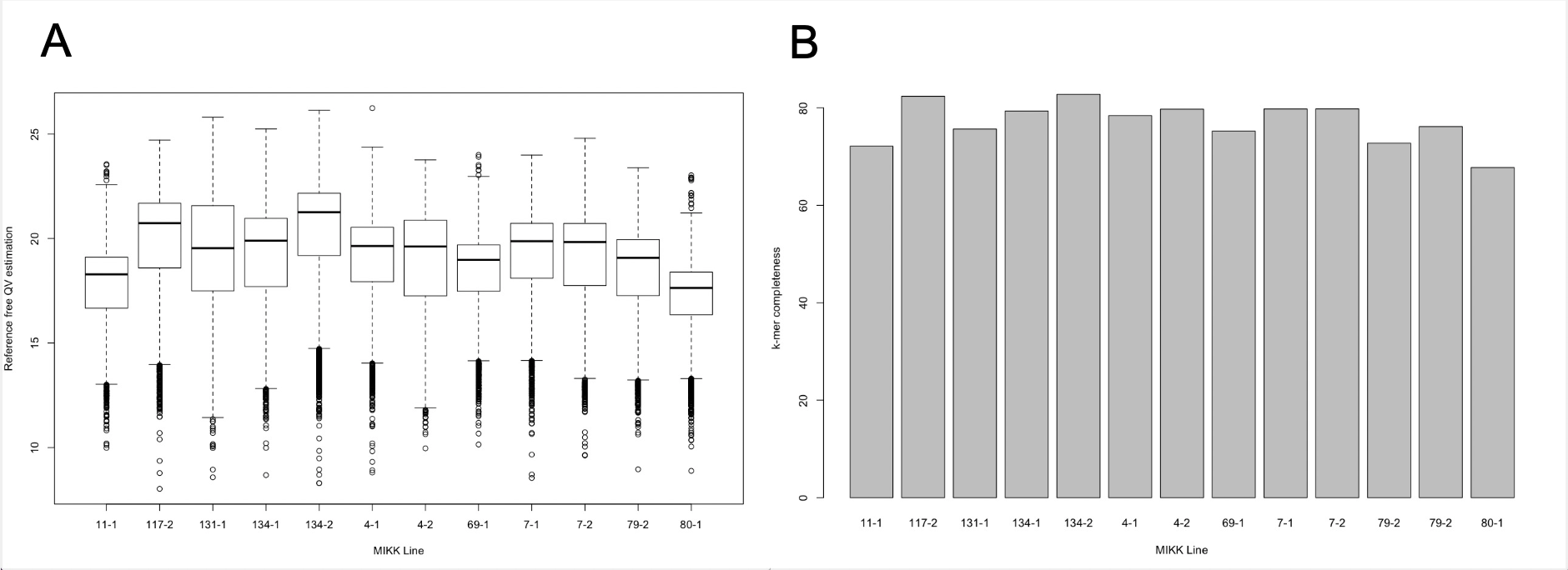
**

**Figure S1**: *Reference free assembly quality assessment using Merqury. A: The QV estimation distribution of all contigs for the 12 MIKK panel assemblies. B: The k-mer completeness for the 12 MIKK panel assemblies.*

*
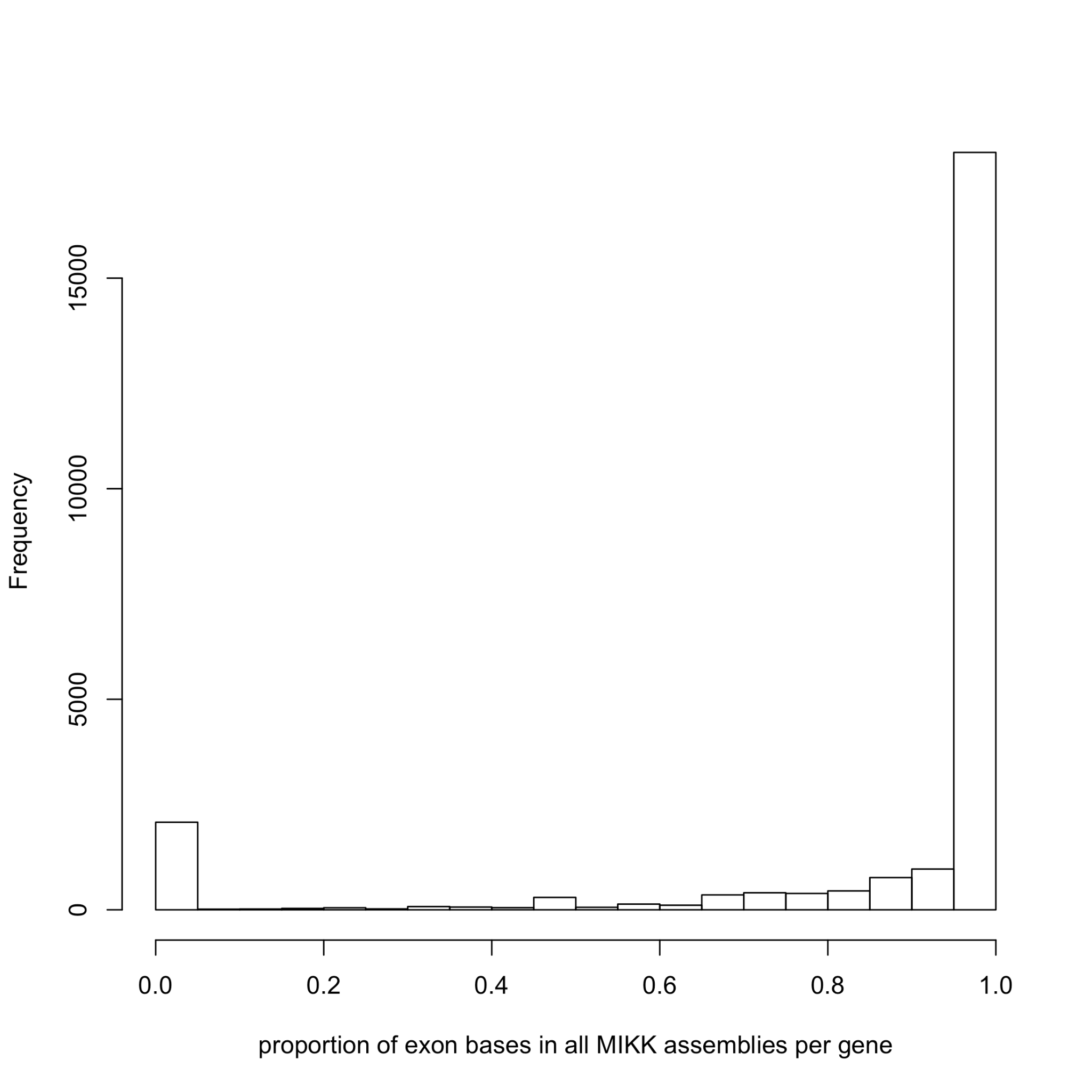
*

**Figure S2**: Histogram showing the proportion of exonic bases covered by all MIKK panel assembles for 24,328 annotated genes from the HdrR reference.

**
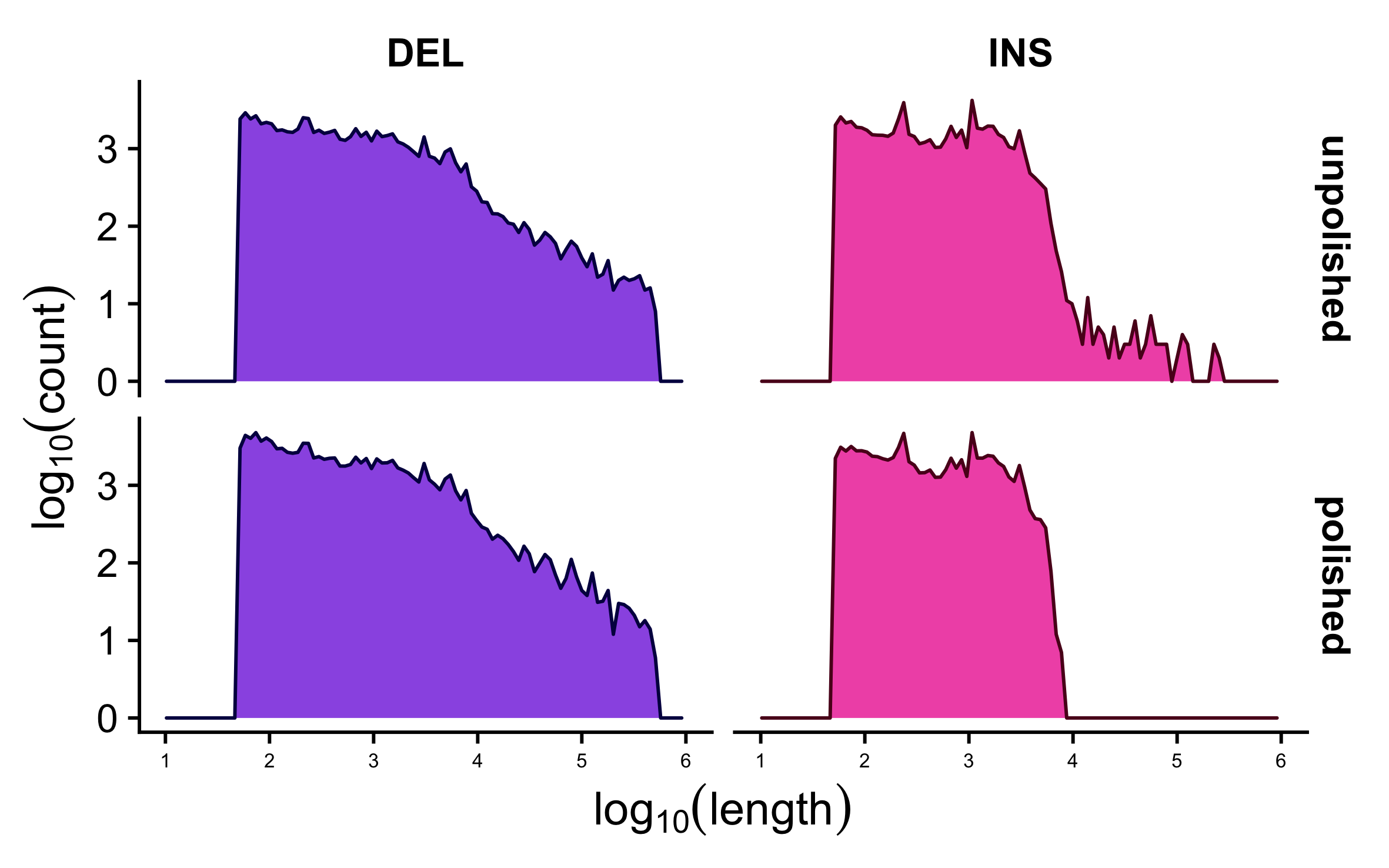
**

**Figure S3**: *log_10_ lengths and counts of deletion (DEL) and insertion (INS) structural variants before and after polishing with SViper.*

**
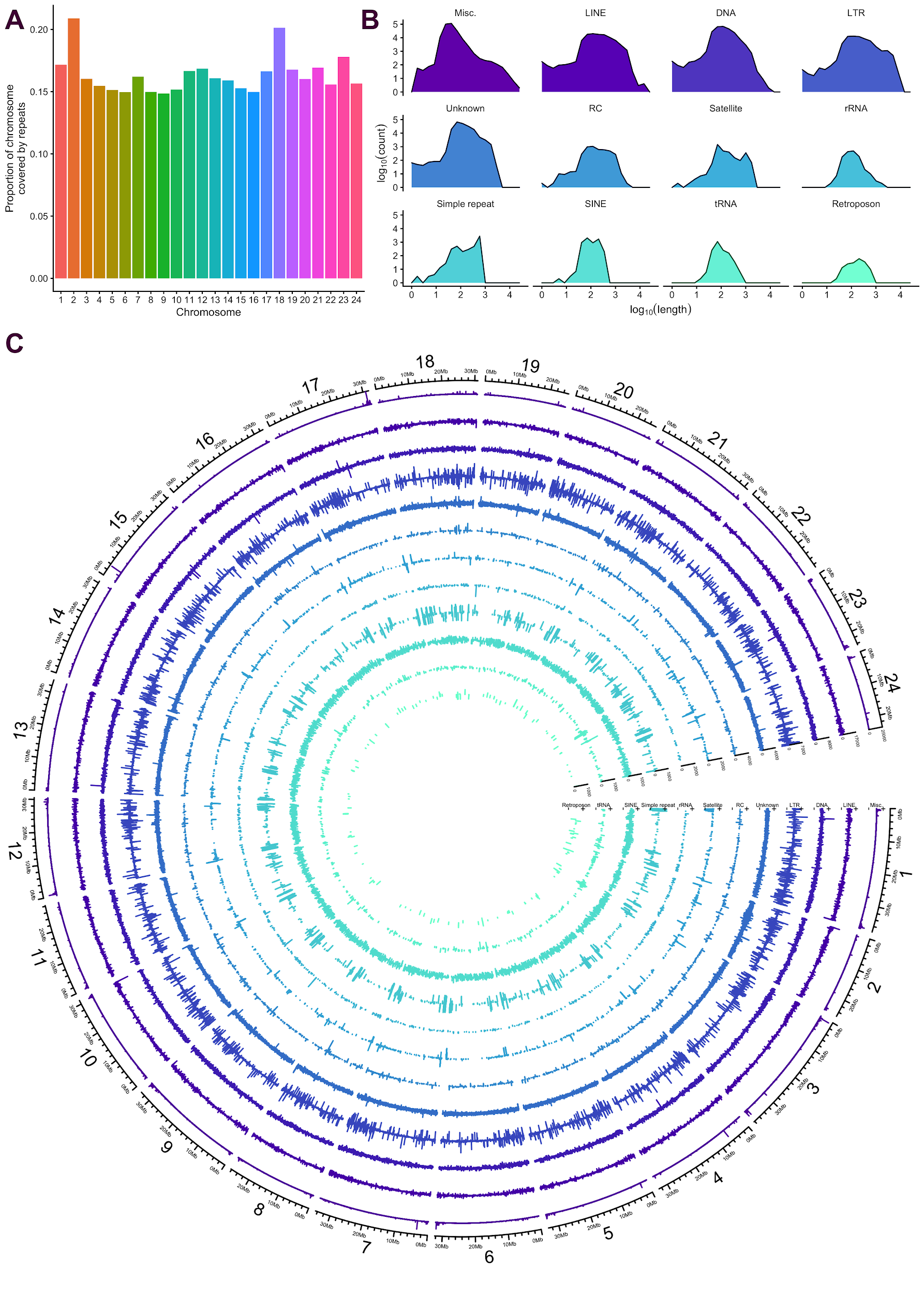
**

**Figure S4**: *Repeat content in the* HdrR *genome based on RepeatMasker results (****Methods****).* ***A****. Proportion of repeat content per-chromosome.* ***B****. log_10_ of repeat lengths and counts per repeat class. “Misc” includes all repeats assigned to their own specific class, for example “(GAG)n” or “(GATCCA)n”.* ***C****. Circos plot showing repeat length (radial axes) by locus (angular axis) and repeat class (track). The code and methods used to generate the figure are set out here:* <https://birneylab.github.io/MIKK_genome_main_paper/20210409_repeats.html>


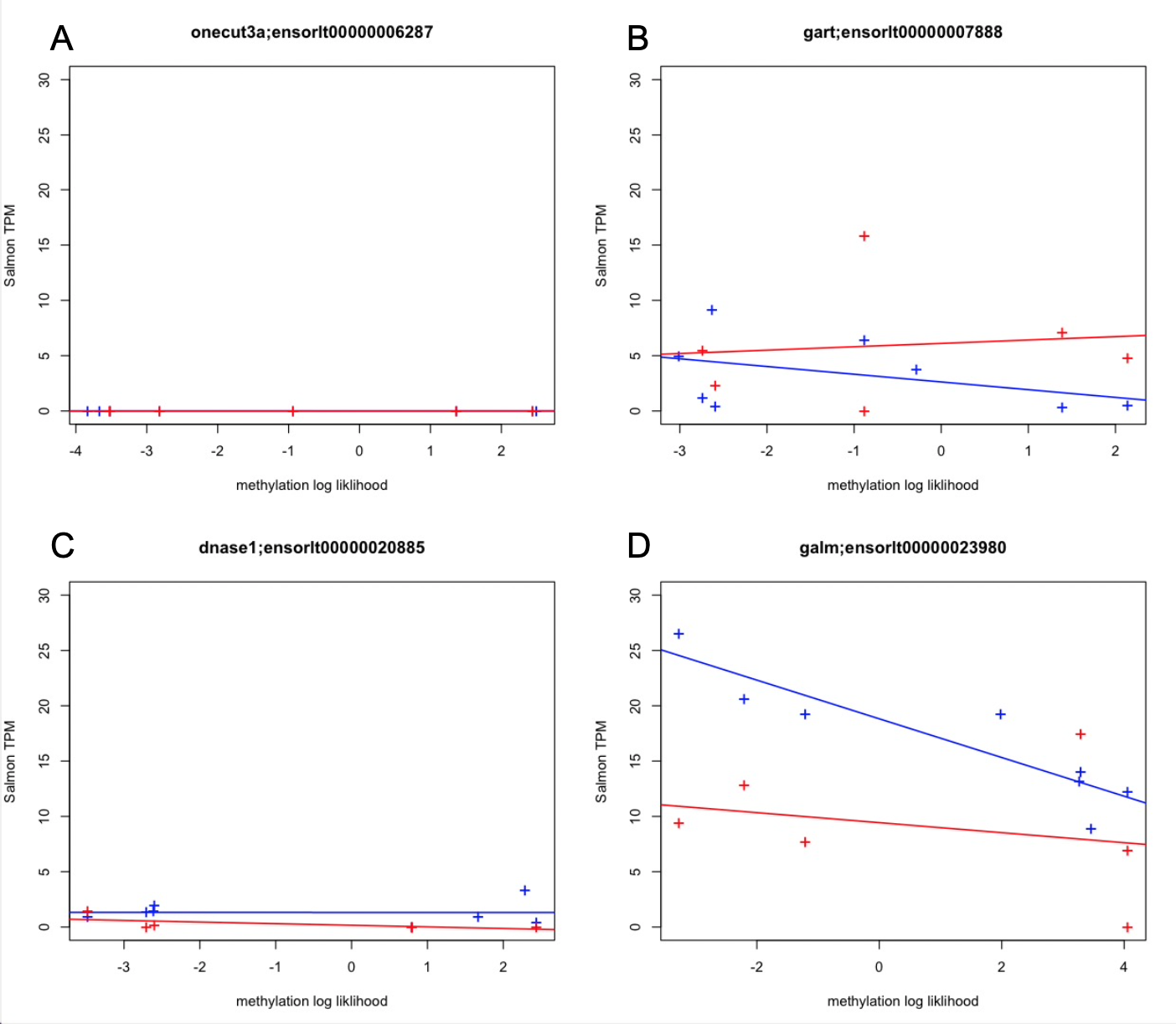


**Figure S5**: *Median methylation log likelihood against Salmon transcripts per million for liver and heart at 4 of the top differentially methylated regions (DMRs).* ***A:*** *Methylation log likelihood against salmon TPM at the onecut3a gene (liver is shown in blue and heart is shown in red),* ***B:*** *Methylation log likelihood against salmon TPM at the gart gene (liver is shown in blue and heart is shown in red),* ***C:*** *Methylation log likelihood against salmon TPM at the dnase1 gene (liver is shown in blue and heart is shown in red),* ***D:*** *Methylation log likelihood against salmon TPM at the galm gene (liver is shown in blue and heart is shown in red).*
