# Supplementary figures and images for: Genomic variations and epigenomic landscape of the Medaka Inbred Kiyosu-Karlsruhe (MIKK) panel
Source: Genome Biol. 2022 Feb 21;23:58. doi: 10.1186/s13059-022-02602-4 (PMC8862245; doi:10.1186/s13059-022-02602-4)

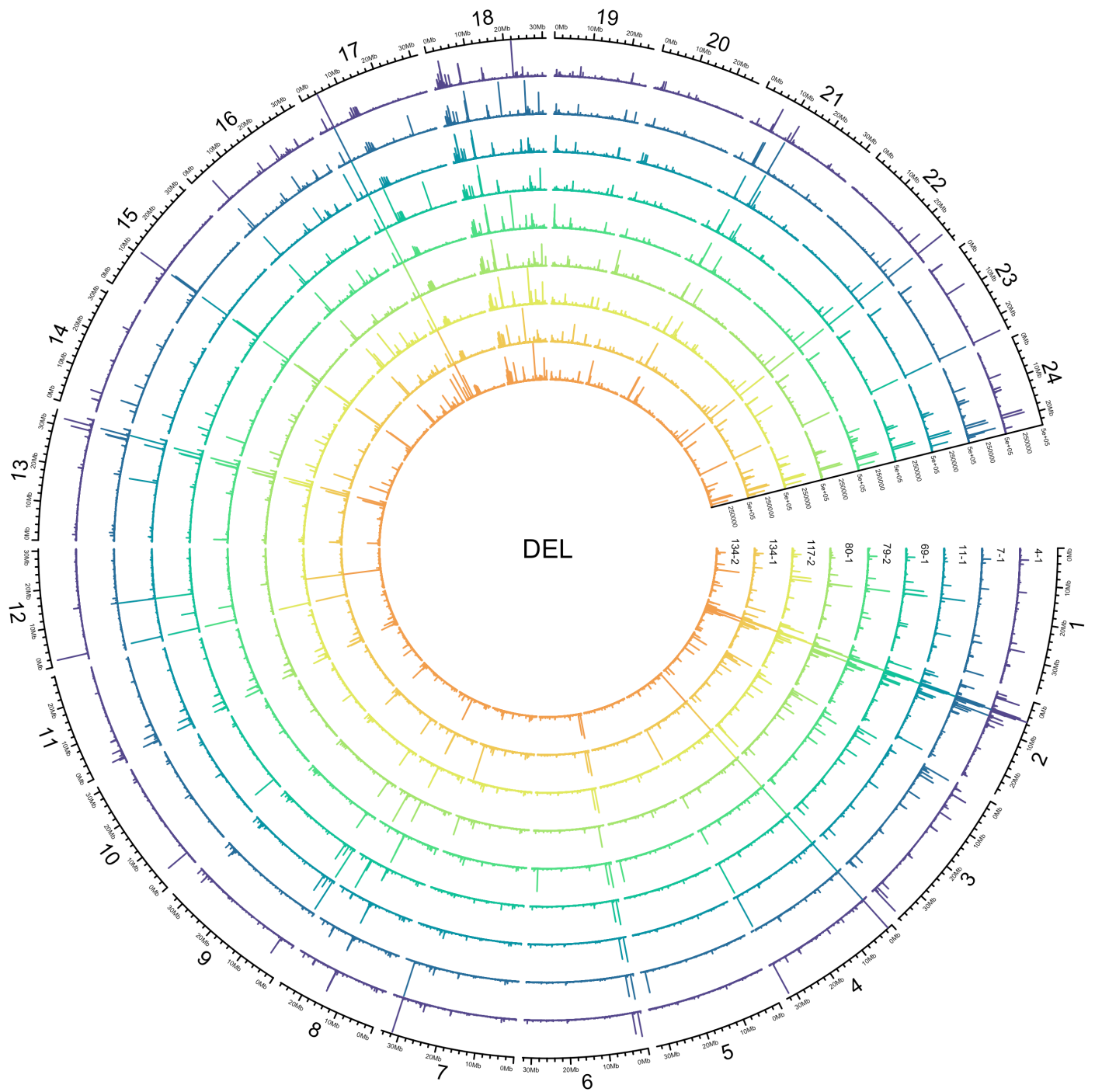

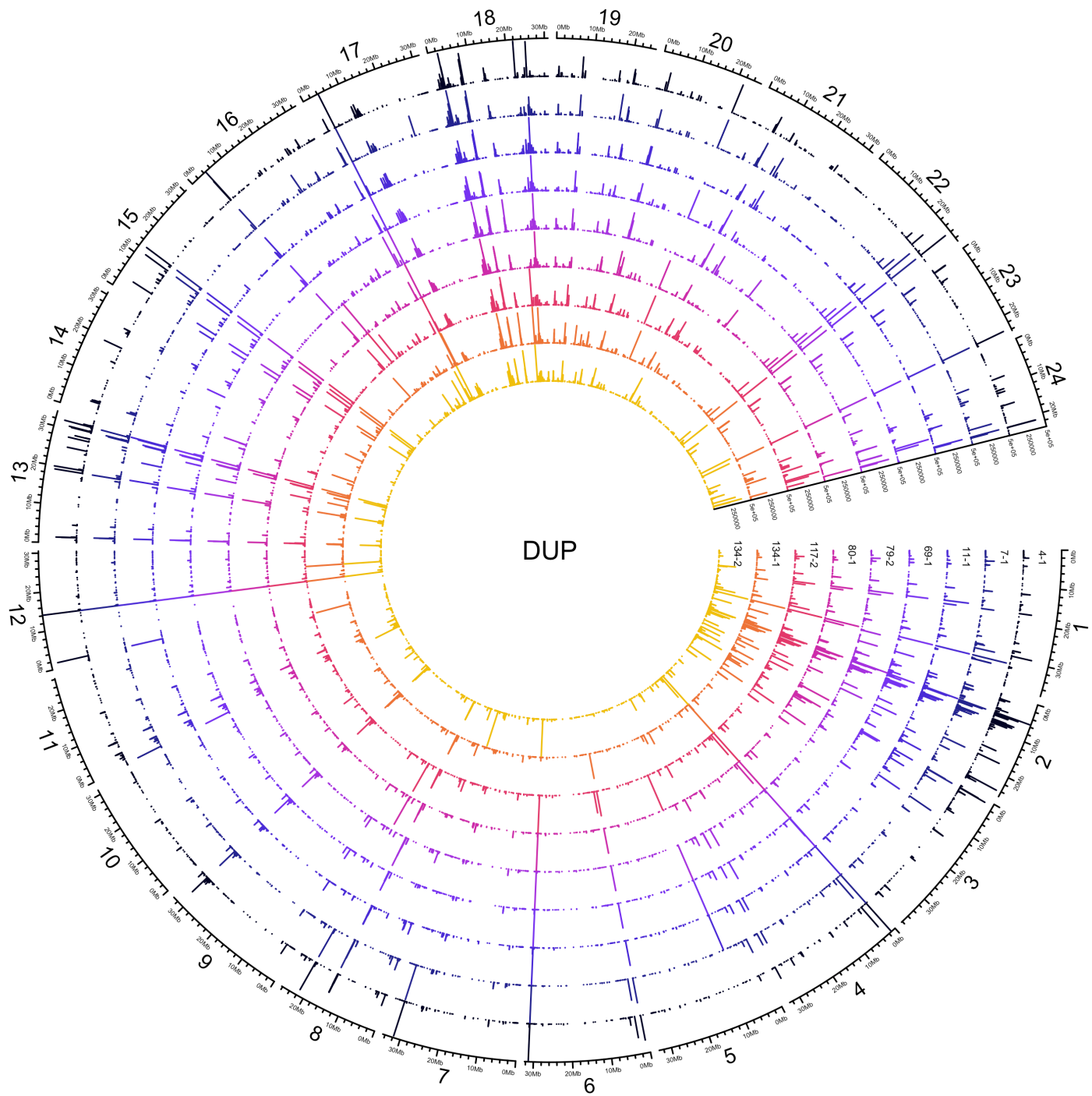

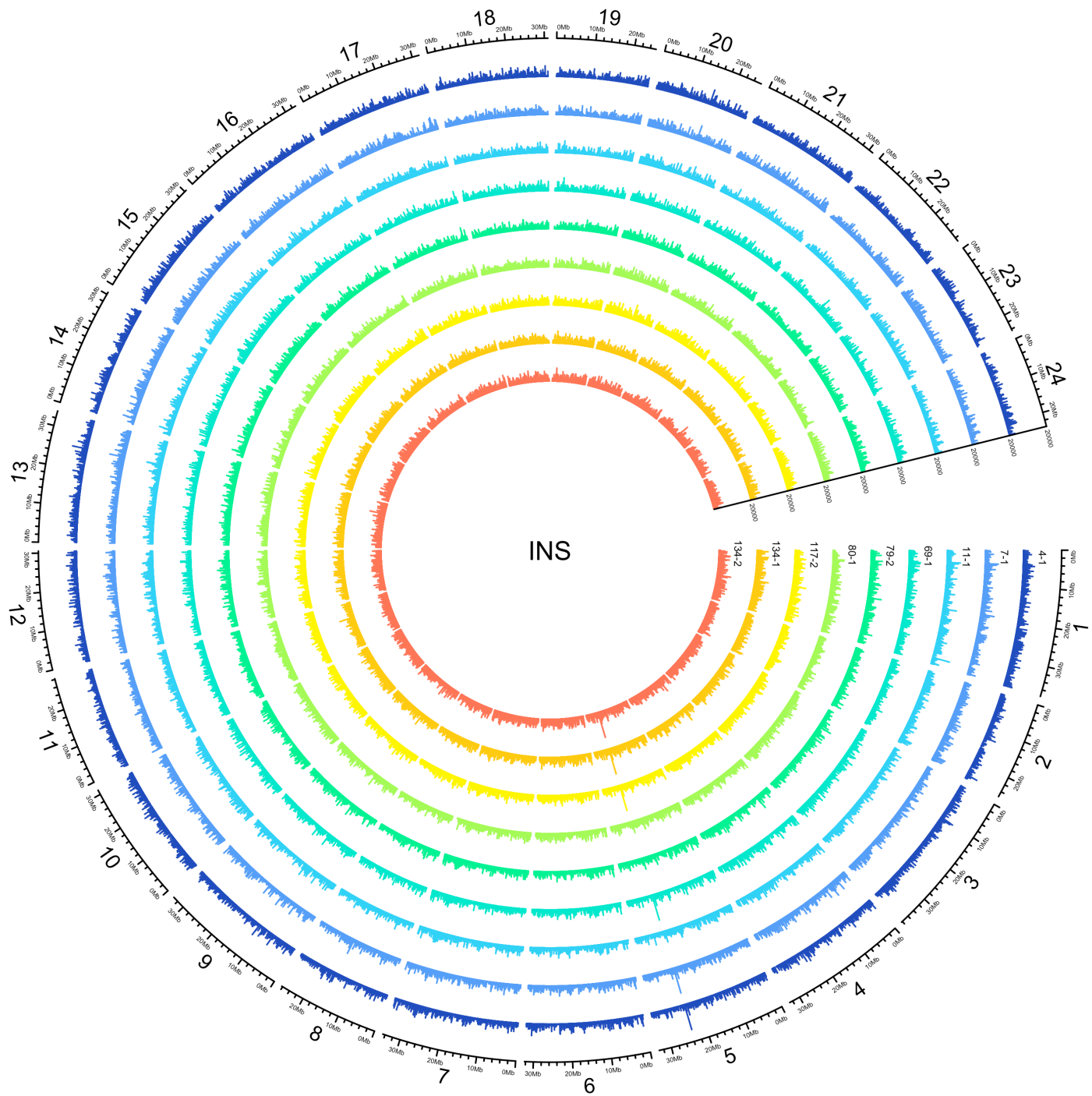

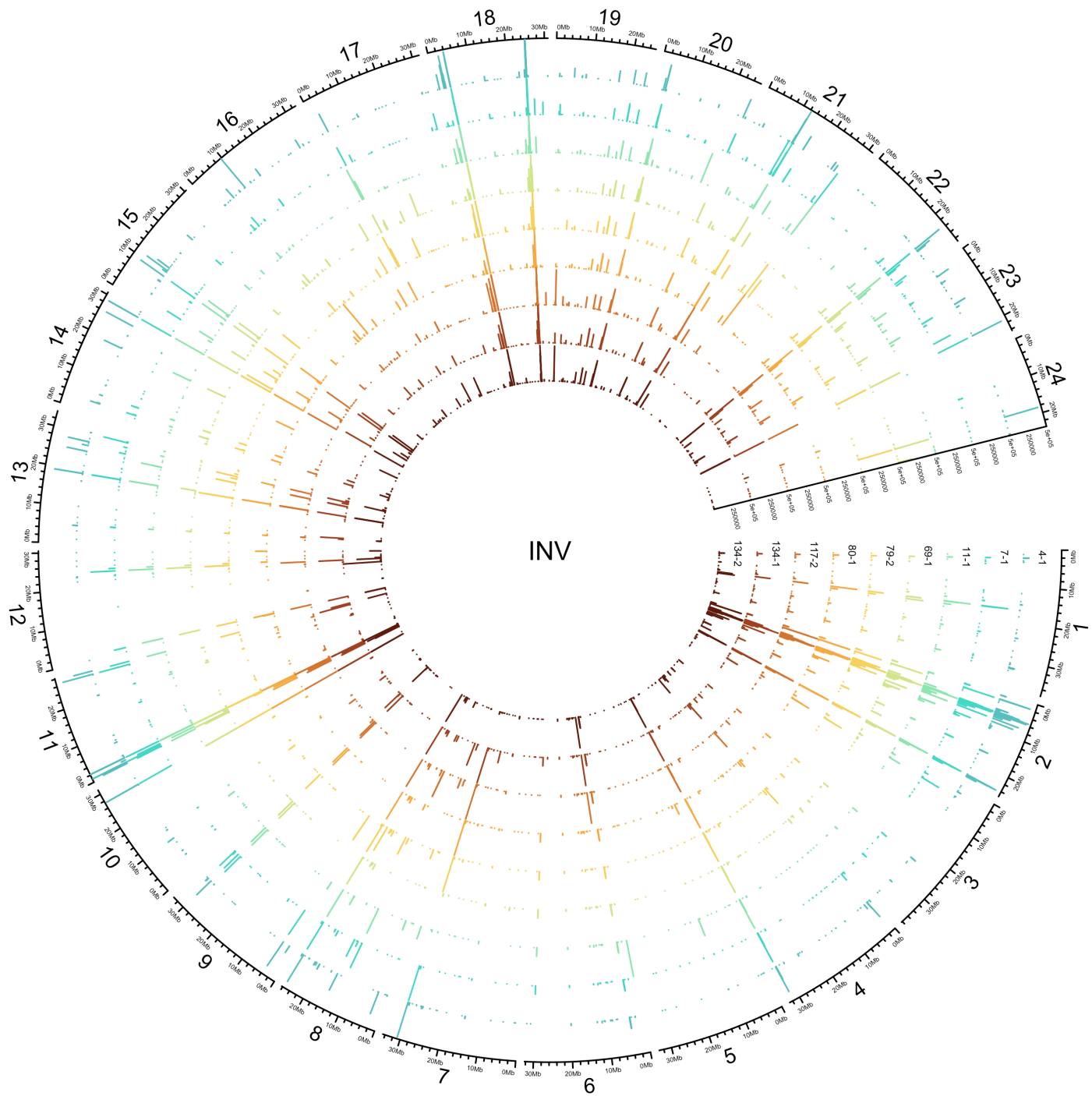

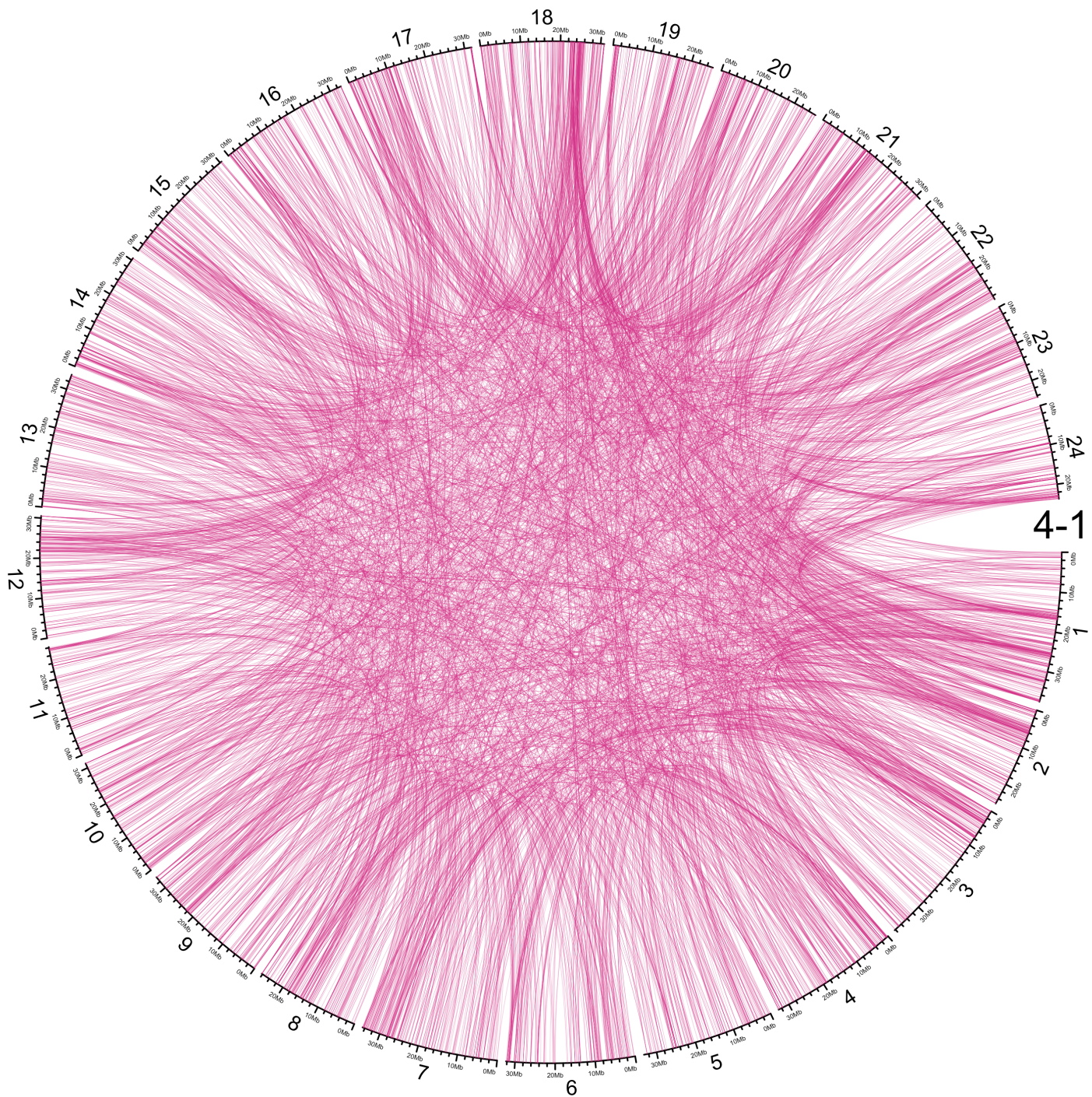

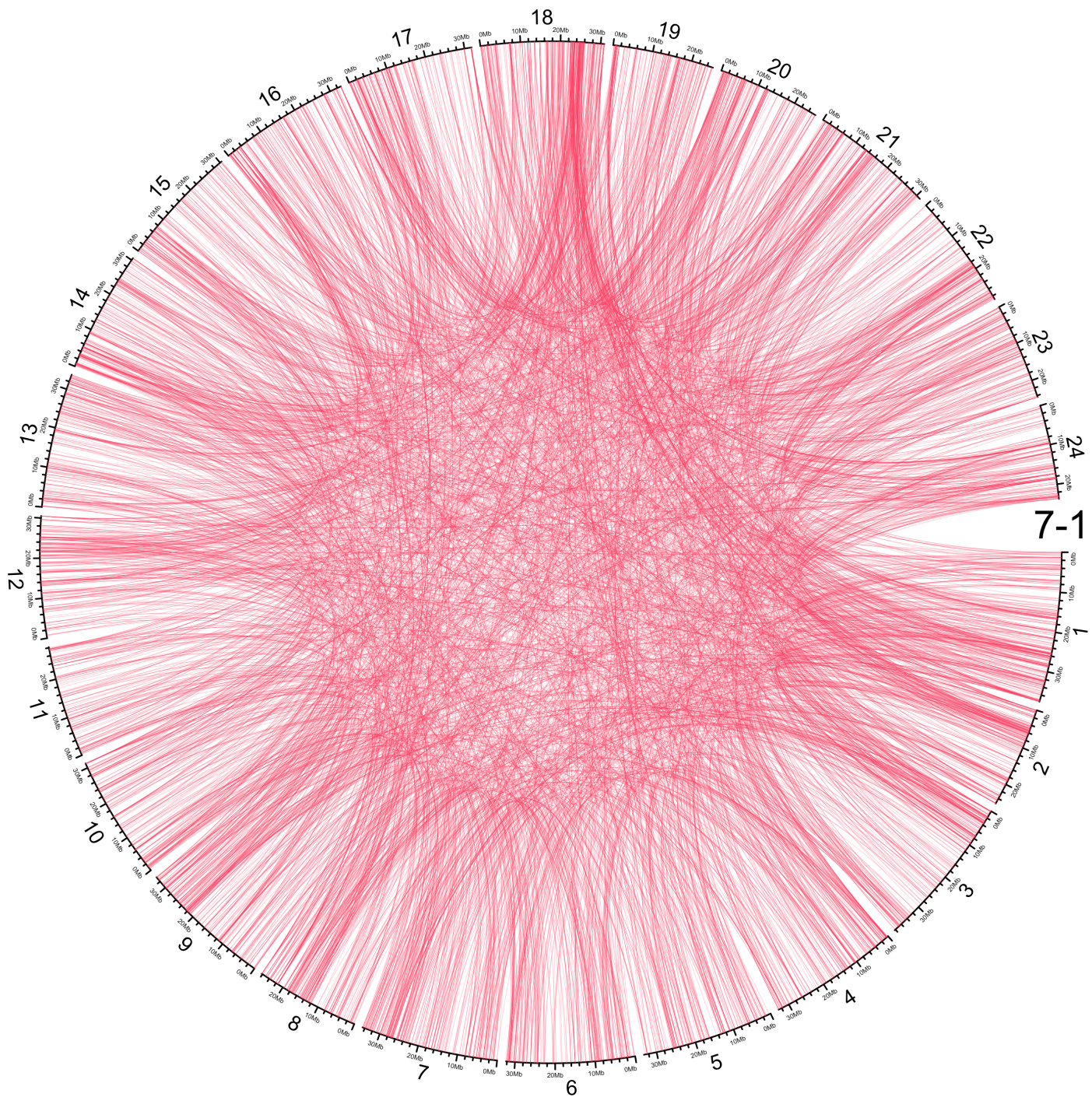

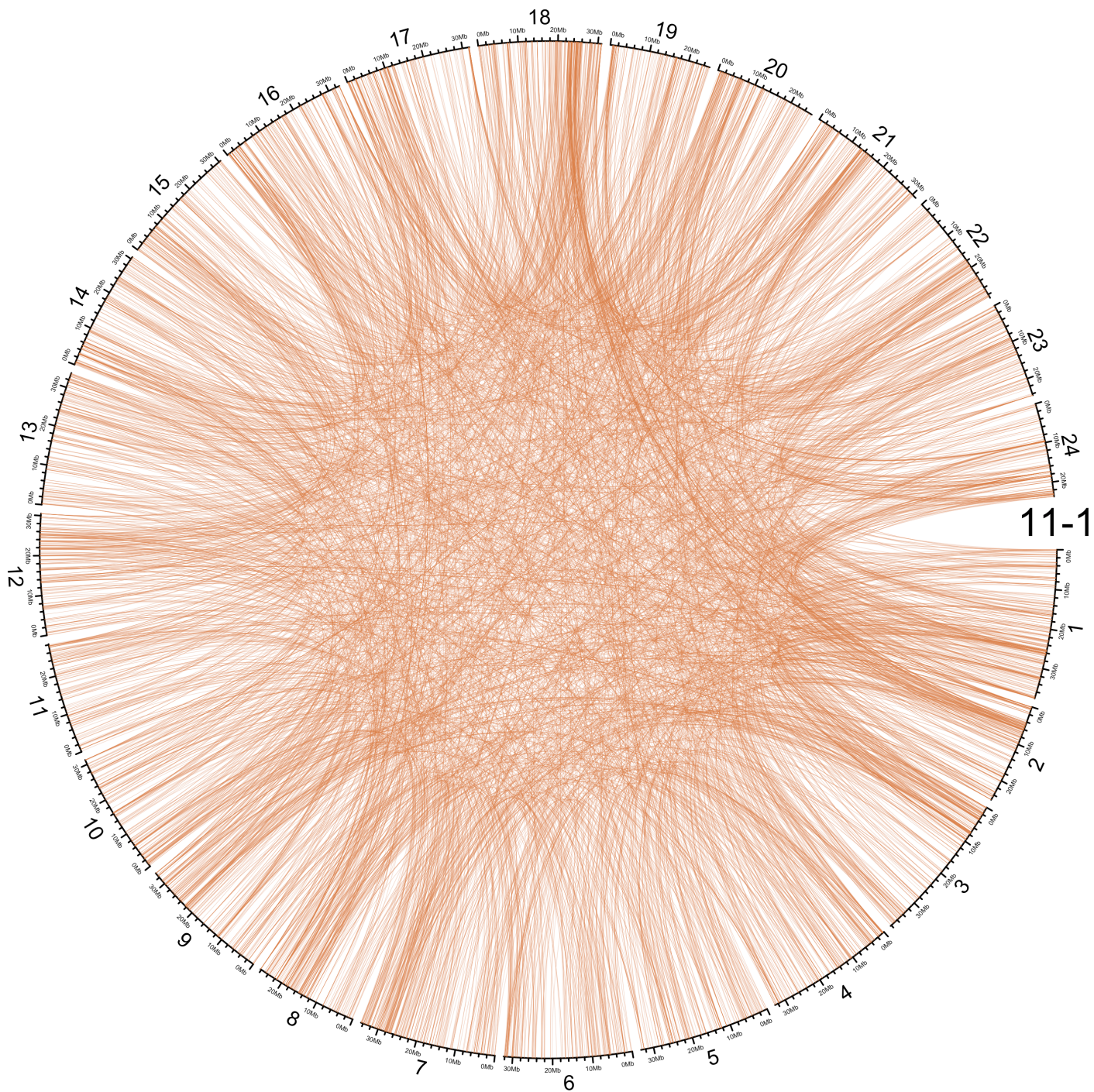

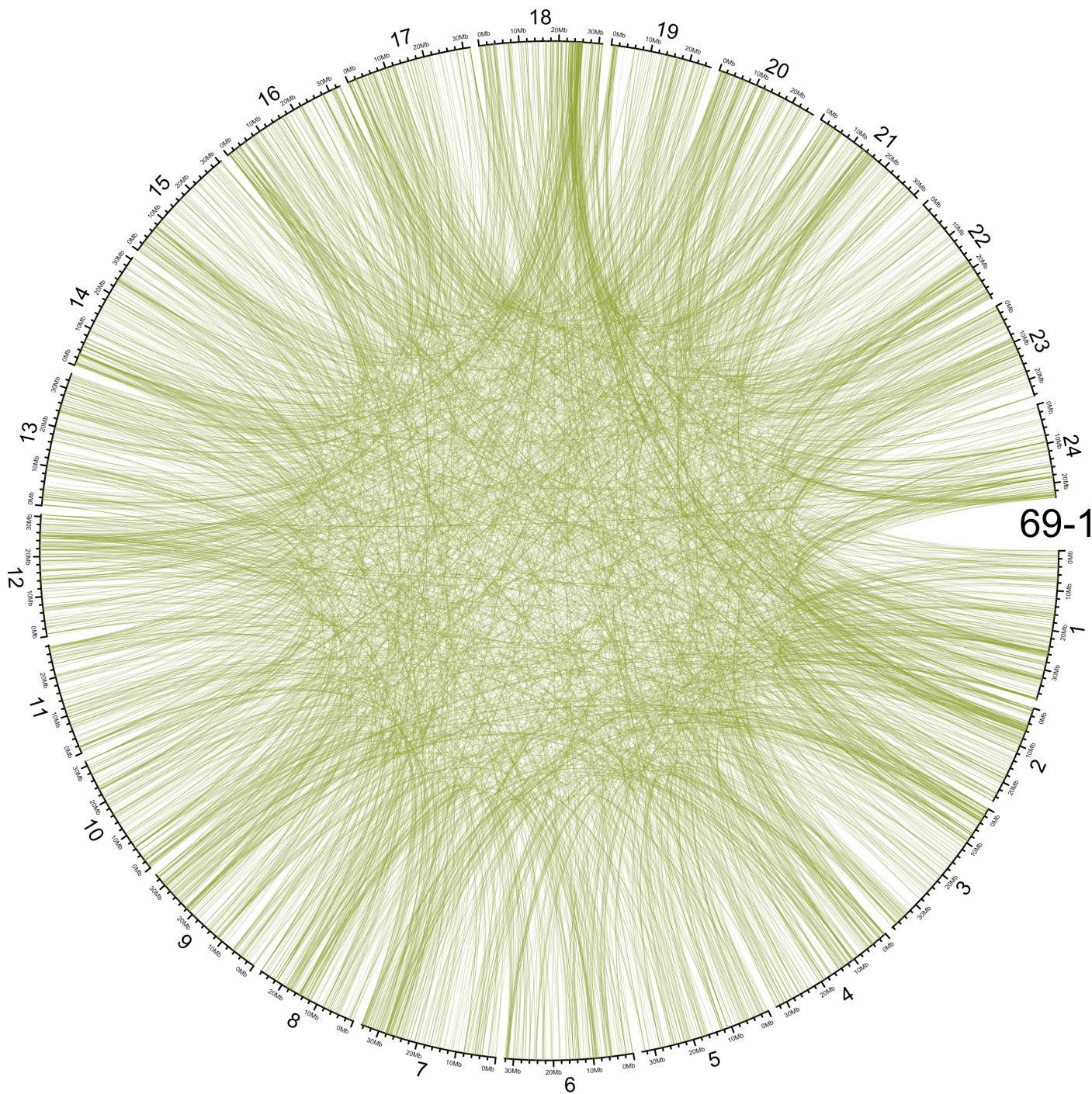

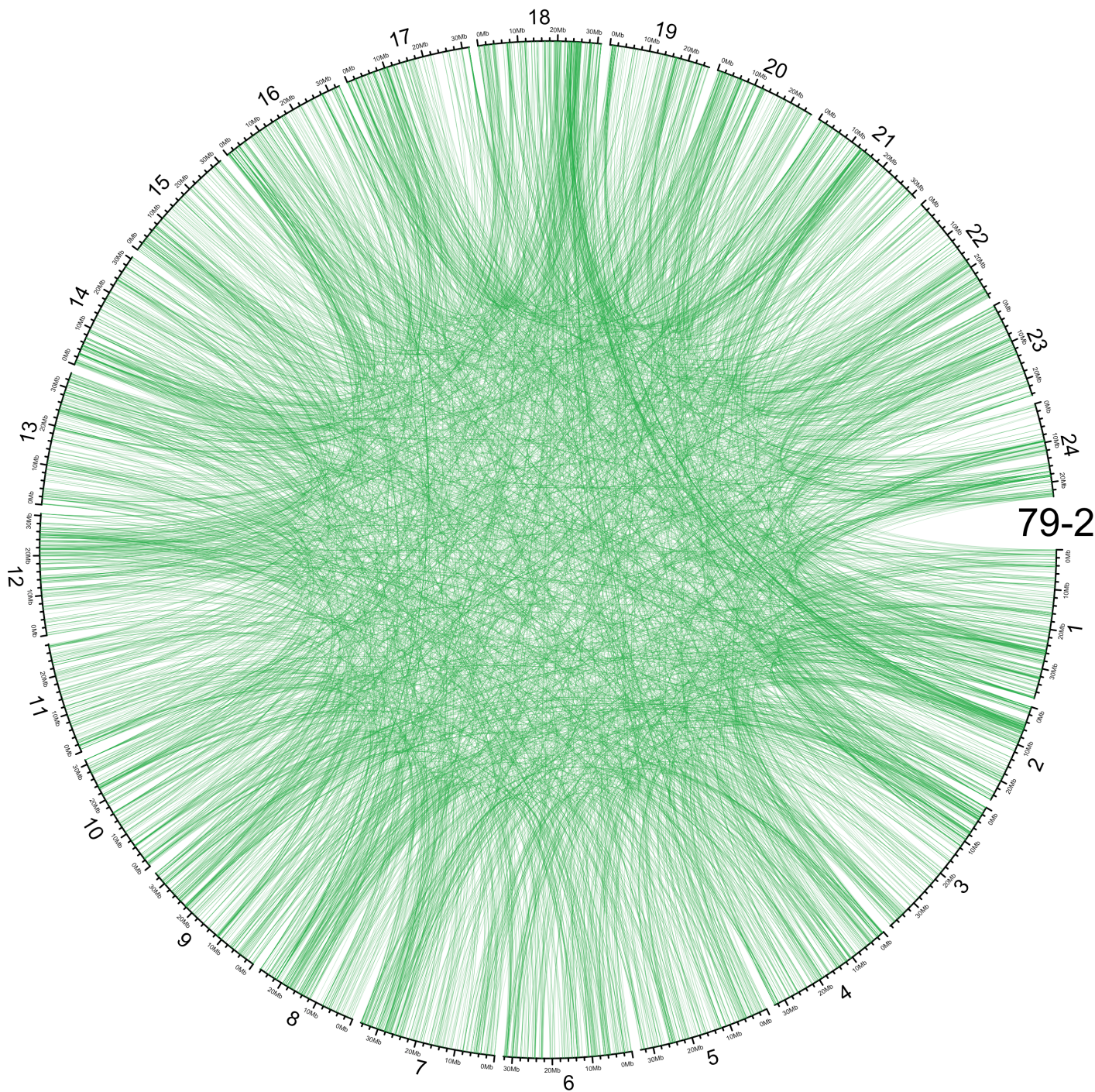

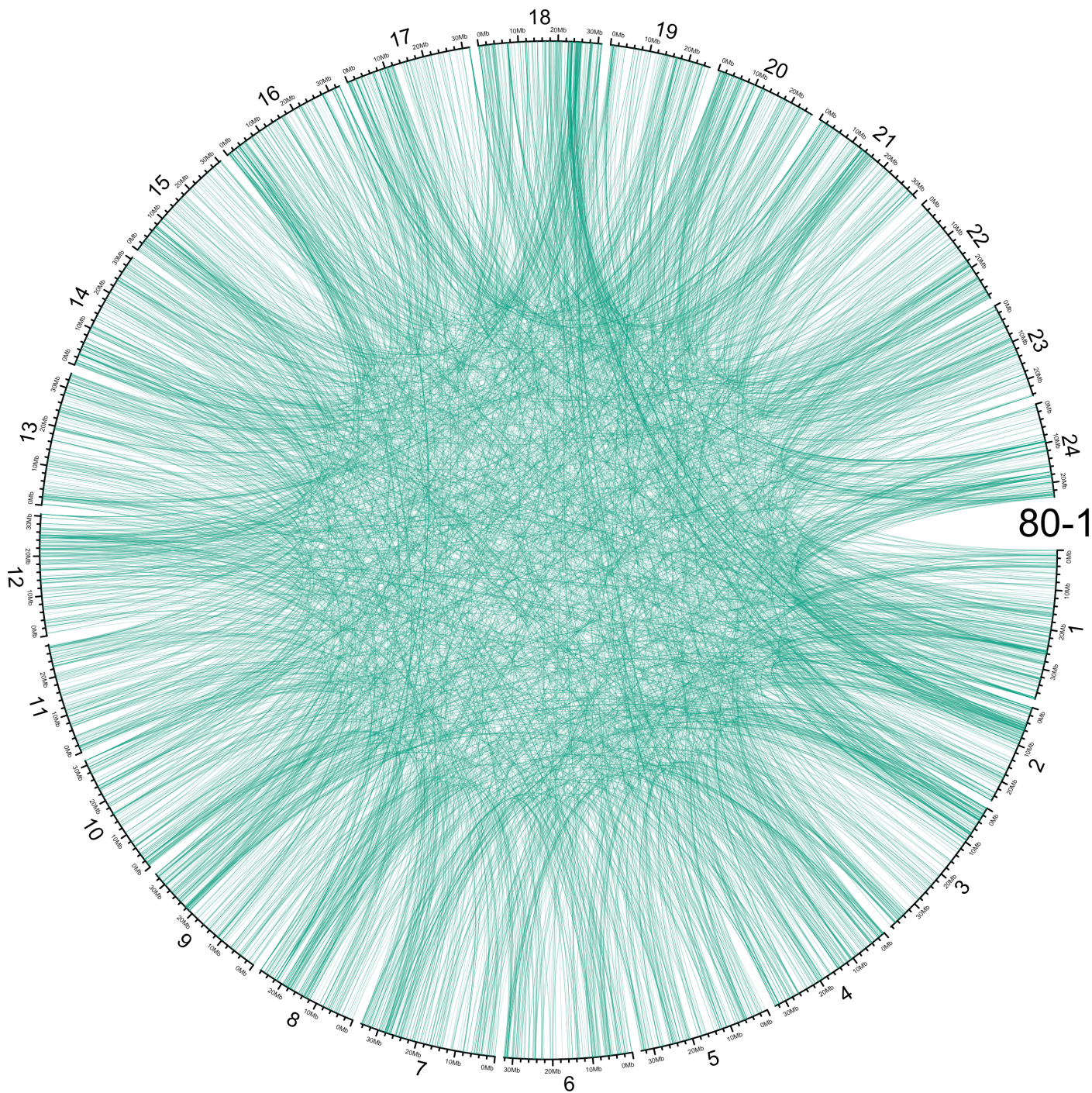

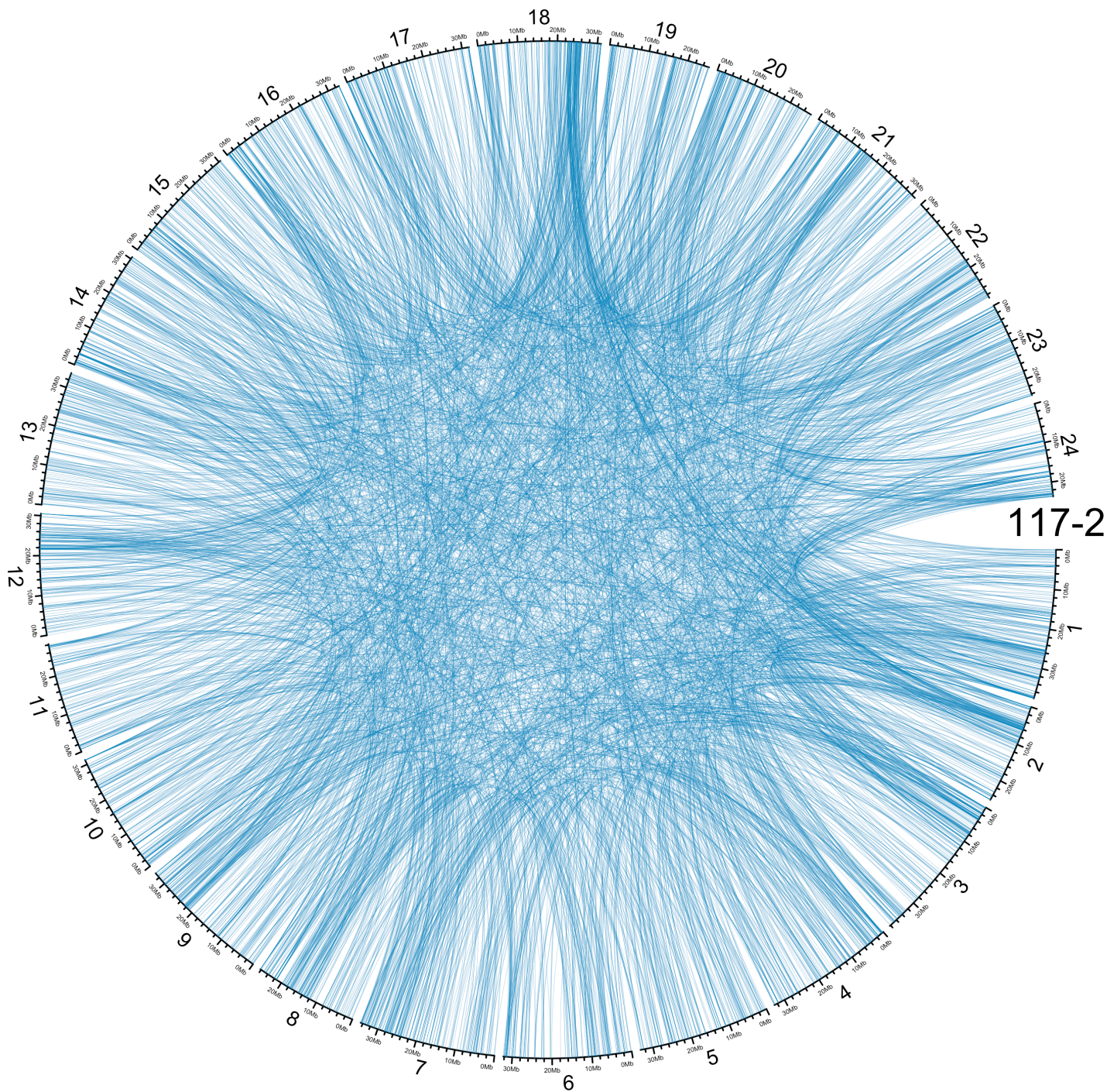

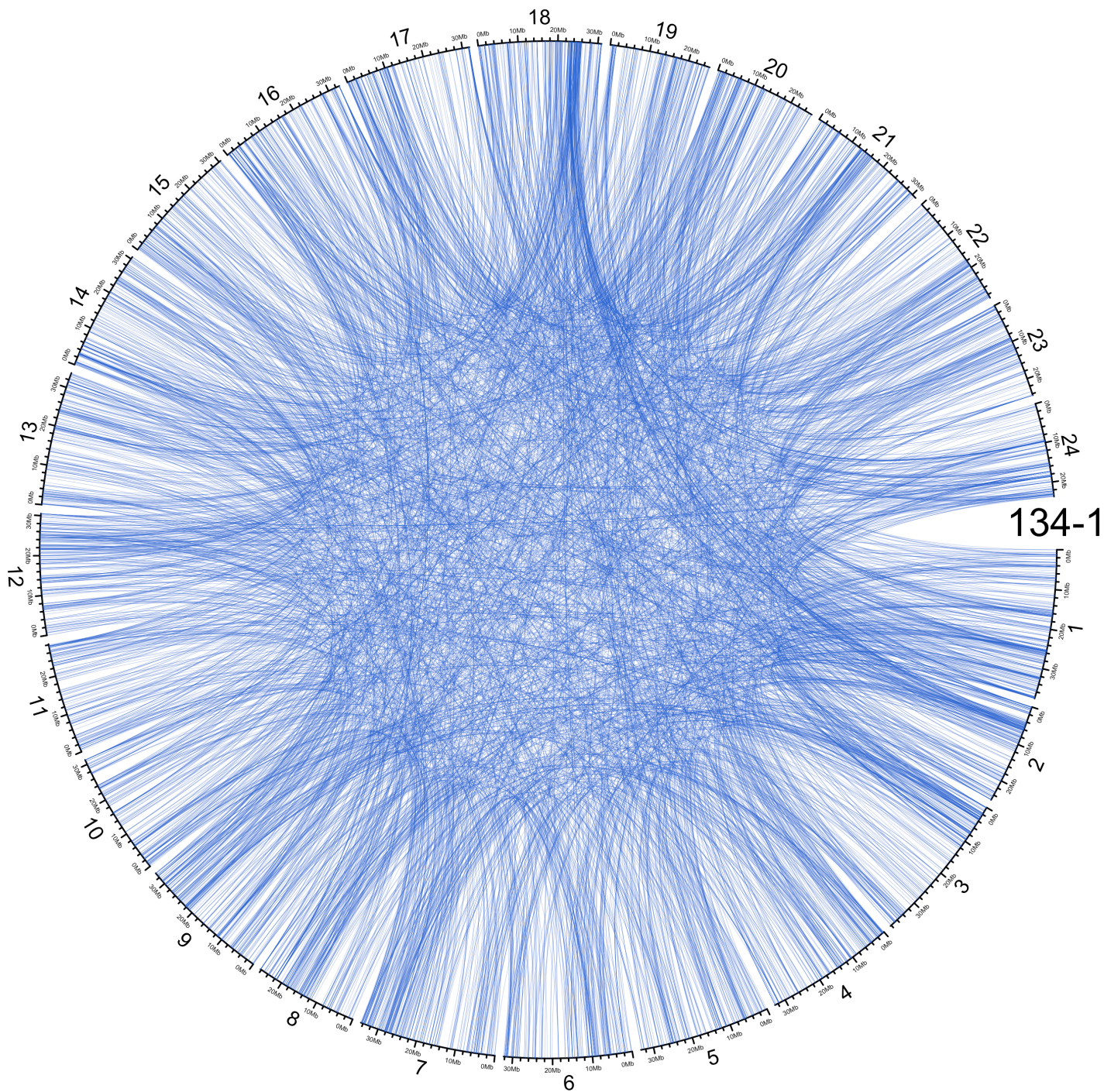

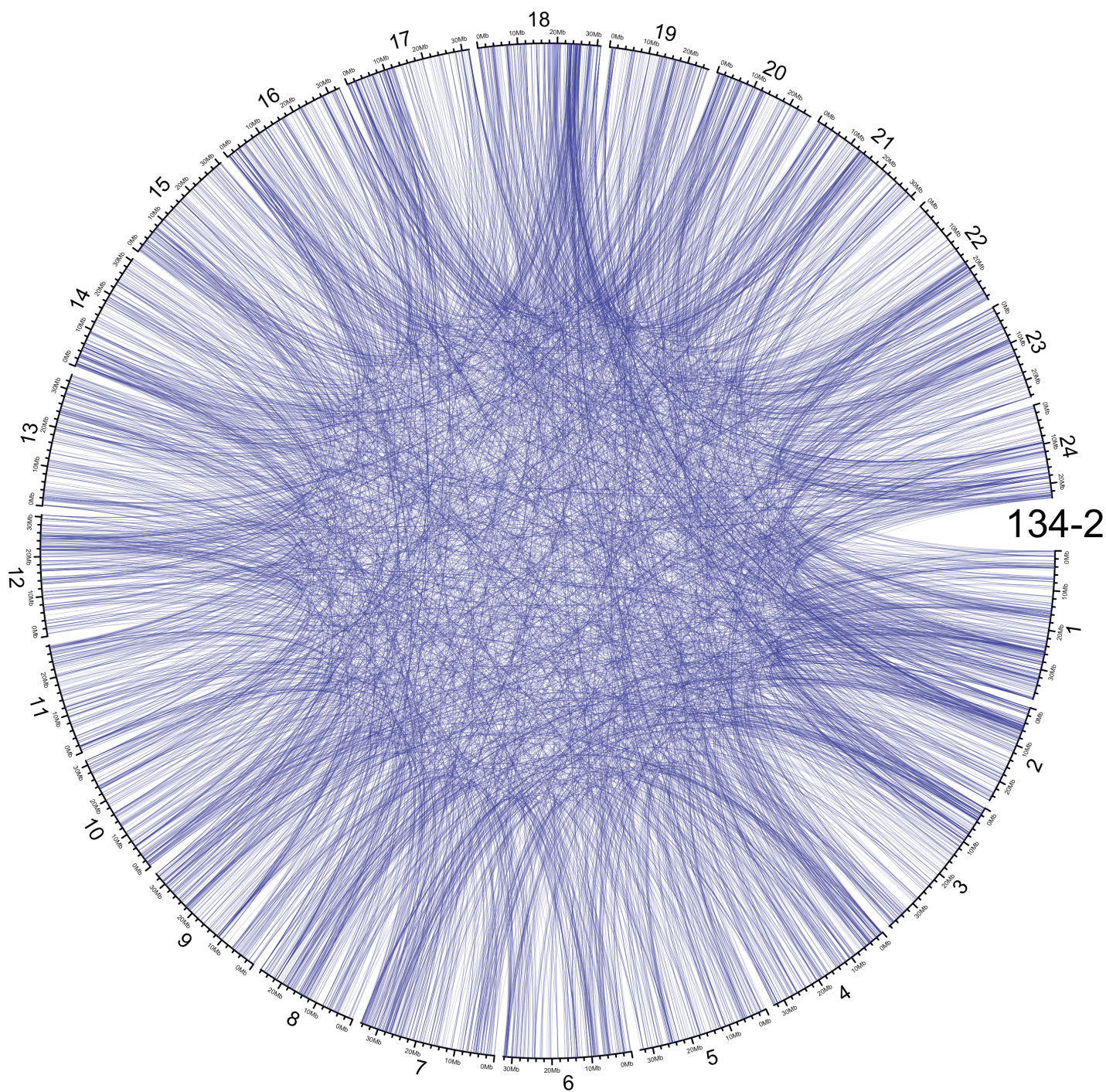

Supplement: Supplementary file 8 — Additional file 8: Figure S6. Circos plots. Circos plots for structural variants (DEL, INS, DUP, INV, TRA) discovered by the reference-anchored approach. [file 13059_2022_2602_MOESM8_ESM.pdf]
