# Supplementary material for: Genomic variations and epigenomic landscape of the Medaka Inbred Kiyosu-Karlsruhe (MIKK) panel
Source: Genome Biol. 2022 Feb 21;23:58. doi: 10.1186/s13059-022-02602-4 (PMC8862245; doi:10.1186/s13059-022-02602-4)
Supplement: Supplementary file 10 — Additional file 10. Review history. [file 13059_2022_2602_MOESM10_ESM.docx]

Review History

**First round of review**

**Reviewer 1**

**Are you able to assess all statistics in the manuscript, including the appropriateness of statistical tests used?**

No, I do not feel adequately qualified to assess the statistics.

**Comments to author:**

Please consider the following questions:

- Are the methods appropriate to the aims of the study, are they well described, and are necessary controls included? If not, please specify what is required.

Yes

- Are the conclusions adequately supported by the data shown? If not, please explain

Yes

- Are sufficient details provided to allow replication and comparison with related analyses that may have been performed? If not, please specify what is required.

Yes

- Does the work represent a significant advance over previously published studies?

Yes

- Is the paper of broad interest to others in the field, or of outstanding interest to a broad audience of biologists?

Yes

In this study, Leger and colleagues investigated genomic variations and epigenomic landscape of the Medaka Inbred Kiyosu-Karlsruhe (MIKK) panel using Oxford Nanopore Technologies (ONT) long read data with the aid of Illumina sequencing. It is well known that structure variants (SVs) are key to phenotypic variation and adaptations in evolution. This study shows that how the ONT long read data could be helpful in identification of "dark" SVs in population genomic studies, which could not be done with Illumina sequencing. The study also shows that variation population graphs are clearly more appropriate at least in medaka, and most likely for all genomes. The study is methodologically sound, with clear aims/outputs. However, I feel that the manuscript could improve by considering the following points before published.

First, I do not feel that the novelty of study has been well highlighted, acoordintg to introduction and discussion. I do not know how this study fills the gap in field and will be a pioneer study in the future.

Second, it is a pioneer study in teleost fishes but not in other organmisms. I do not feel that the writing gives adequate credits to other similar pioneer studies.

Third, the authors said that "Standard linear reference alignment approaches can only partially handle large and complex structural variation, and a considerable fraction of genetic variation between individuals is masked from sight.", so how many SVs identified in this study are such cases which are not identified with Illumina sequencing in their back to back companion paper.

Forth, any overlaps between results of pangenome variation graphs and SVs in the reference-anchored manner?

Fifth, in both pangenome variation graphs and SVs in the reference-anchored manner, I found a deletion bias from numbers to length. I am not sure if this is a true genome evolution trend or bias due to methodolgy. This should be justified.

Finally, the ms seems to be written in a rush. For example, Table SX (P27 L10) is missing, "1)" should be added after "supported" (P27 L12) , a comma is missing after "lines" (P27 L13).

**Reviewer 2**

**Are you able to assess all statistics in the manuscript, including the appropriateness of statistical tests used?**

No, I do not feel adequately qualified to assess the statistics.

**Comments to author:**

Please consider the following questions:

- Are the methods appropriate to the aims of the study, are they well described, and are necessary controls included? If not, please specify what is required.

- Are the conclusions adequately supported by the data shown? If not, please explain

- Are sufficient details provided to allow replication and comparison with related analyses that may have been performed? If not, please specify what is required.

- Does the work represent a significant advance over previously published studies?

- Is the paper of broad interest to others in the field, or of outstanding interest to a broad audience of biologists?

This is a good research of pan-genome by using Oxford Nanopore Technologies long read data. With the new data, "dark variations" were able to be uncovered. In this manuscript, authors worked on the genome assembly using wtdbg2, built the pan-genome graph using minigraph and found the variations among the MIKK panel lines. Overall, it was a meaningful work. However, there are some points needed to be improved.

1.It is confused that authors concluded "Thus we present here a for genome analysis that has the potential to become a standard approach for population studies." as well as the Abstract section. However, I did not find novel method for pan-genome? The Method section, the pycoSnake pipeline was mentioned, but it did not include the method of graphing pan-genome. Please make it clear of "methodology" or carefully correct the statement.

2.In this manuscript, authors did not talk about the core genes or comparison of functional genes, which is essential for pan-genome analysis.

3.12 MIKK lines were selected from the 80 stable lines except 7-1, 131-1 and 134-2. What is the criterion of this selection? And why these 12 lines even include sibling lines?

4.In page 2 line 9-11 of the "Abstract" section, authors describe "Nanopore sequencing allowed us to identify a much greater variety of high-quality structural variants compared with Illumina sequencing", but there was no result about the comparision with Illumina sequencing other than polished SVs identified by Nanopore sequencing with Illumina short reads. Please give citation or data proof.

5.In line 12-15 of "Abstract" section, there are no results of linear reference alignment approach, so the "novel differences" and "additional MIKK panel-specific genomic content" can not be concluded.

6.Authors discovered structural variations both with graph and reference-anchored manner, but the results seem disconnected. In this case, it could not make sense. Please merge or compare the results of the two sections.

7.In page 6 line 16, the quast result is absent. Besides, add the BUSCO result on genome quality. Please give the assessment of genome assemblies using Merqury.

8.In Fig. 2A, "the possible reference deletion" should mean that the segment is deleted in reference HDRR, why it still belongs to reference (colored with HDRR)? Please make the illustration clear.

9.The labels in x axis of Fig. 2F is incomplete.

10.In page 12 line 15, the result of CNVs is absent.

11.In page 14 line 20-21, what about the function of these four "interesting" genes? Moreover, whether their expressions are changed owing to DMR?

12.In page 12 line 12 "similar to many human studies", please add reference.

**Authors Response**

**Point-by-point responses to the reviewers’ comments:**

Reviewer #1:

In this study, Leger and colleagues investigated genomic variations and epigenomic landscape of the Medaka Inbred Kiyosu-Karlsruhe (MIKK) panel using Oxford Nanopore Technologies (ONT) long read data with the aid of Illumina sequencing. It is well known that structure variants (SVs) are key to phenotypic variation and adaptations in evolution. This study shows that how the ONT long read data could be helpful in identification of "dark" SVs in population genomic studies, which could not be done with Illumina sequencing. The study also shows that variation population graphs are clearly more appropriate at least in medaka, and most likely for all genomes. The study is methodologically sound, with clear aims/outputs. However, I feel that the manuscript could improve by considering the following points before published.

First, I do not feel that the novelty of study has been well highlighted, acoordintg to introduction and discussion. I do not know how this study fills the gap in field and will be a pioneer study in the future.

*Response: We have added some more context and references into both the introduction and discussion highlighting that one main aspects of this study is in showcasing some of the benefits of moving away from the linear reference and providing clear examples of how these types of variation can be interpreted in context (in this case variation in medaka MIKK panel).*

Second, it is a pioneer study in teleost fishes but not in other organmisms. I do not feel that the writing gives adequate credits to other similar pioneer studies.

*Response: We apologize for this and have made more extensive reference to previous work in other organisms and cite multiple more recent methodological advancements in graph based genome representations and variant analysis.*

Third, the authors said that "Standard linear reference alignment approaches can only partially handle large and complex structural variation, and a considerable fraction of genetic variation between individuals is masked from sight.", so how many SVs identified in this study are such cases which are not identified with Illumina sequencing in their back to back companion paper.

*Response: We have added a section to compare the SVs that were detectable using ONT data via the linear reference alignment approach against the graph and show an improvement in the number and resolution of SV’s detected when using the graph based methods (and in particular for novel insertions). We have removed all reference to improved detection of SV’s from ONT compared to Illumina.*

Forth, any overlaps between results of pangenome variation graphs and SVs in the reference-anchored manner?

*Response: We have added a section at the end of Results “Structural variation and breakpoint mapping in the MIKK panel” looking at the overlap between the SV’s we described from the graph (novel insertions and deletions) against those detected using the reference anchored approach and found that SV’s from the graph where more complete and had better mapping resolution.*

Fifth, in both pangenome variation graphs and SVs in the reference-anchored manner, I found a deletion bias from numbers to length. I am not sure if this is a true genome evolution trend or bias due to methodolgy. This should be justified.

*Response: This is an interesting observation and we think that this bias is due to methodology as the ability to detect insertions drops sharply for those larger than 10,000bp. The median read length across all samples is approximately three quarters of this, suggesting that the length of resolvable insertions is likely to be directly proportional to the sequencing read length. We have added a point to the discussion concerning this observation.*

Finally, the ms seems to be written in a rush. For example, Table SX (P27 L10) is missing, "1)" should be added after "supported" (P27 L12) , a comma is missing after "lines" (P27 L13).

*Response: We apologize for these error - we have carefully gone through the entire document to correct these types of mistakes.*

Reviewer #2:

This is a good research of pan-genome by using Oxford Nanopore Technologies long read data. With the new data, "dark variations" were able to be uncovered. In this manuscript, authors worked on the genome assembly using wtdbg2, built the pan-genome graph using minigraph and found the variations among the MIKK panel lines. Overall, it was a meaningful work. However, there are some points needed to be improved.

1.It is confused that authors concluded "Thus we present here a for genome analysis that has the potential to become a standard approach for population studies." as well as the Abstract section. However, I did not find novel method for pan-genome? The Method section, the pycoSnake pipeline was mentioned, but it did not include the method of graphing pan-genome. Please make it clear of "methodology" or carefully correct the statement.

*Response: We apologize for this - that statement was indeed a bit misleading - we have changed it to make it clear that the methodology presented here is for downstream analysis of pangenome reference graphs. We have checked the methods section and found that we did describe and reference the use of minigraph2 for building the pan genome graphs - methods sections “De novo assembly of MIKK panel genomes“, Variation pangenome graph assembly” and “Graph alignment and segment usage analysis”.*

2.In this manuscript, authors did not talk about the core genes or comparison of functional genes, which is essential for pan-genome analysis.

*Response: We have added a description in “Results: Pangenome graph assembly and read alignment” of an assessment into MIKK panel graph segment usage in relation to the HdrR reference gene content. Overall we found that 92% of annotated gene coordinates are covered by graph segments used by at least one MIKK line and that the majority (87%) of coding exons within these genes are fully covered by a single graph segment.*

3.12 MIKK lines were selected from the 80 stable lines except 7-1, 131-1 and 134-2. What is the criterion of this selection? And why these 12 lines even include sibling lines?

*Response: We have added a short sentence in methods to describe this selection - primarily the selection was based on DNA availability but additionally we choose to select samples displaying homozygosity levels across the range of MIKK panel samples and additionally ensured the inclusion of 3 sibling lines to allow a comparison of more closely related lines.*

4.In page 2 line 9-11 of the "Abstract" section, authors describe "Nanopore sequencing allowed us to identify a much greater variety of high-quality structural variants compared with Illumina sequencing", but there was no result about the comparision with Illumina sequencing other than polished SVs identified by Nanopore sequencing with Illumina short reads. Please give citation or data proof.

*Response: We have removed all reference to improved detection of SV’s using ONT vs. Illumina data and have rather focussed on the comparison between SV’s detected using the graph and linear alignment approaches.*

5.In line 12-15 of "Abstract" section, there are no results of linear reference alignment approach, so the "novel differences" and "additional MIKK panel-specific genomic content" can not be concluded.

*Response: We have carefully reworded this sentence and have provided results using a linear reference alignment approach - Results “Structural variation and breakpoint mapping in the MIKK panel”.*

6.Authors discovered structural variations both with graph and reference-anchored manner, but the results seem disconnected. In this case, it could not make sense. Please merge or compare the results of the two sections.

*Response: We have added sections in both the results and discussion that compares the concordance between the high-quality graph-based alternative alignment paths and large-scale deletions, and the deletions and insertions discovered through the reference-based approach.*

7.In page 6 line 16, the quast result is absent. Besides, add the BUSCO result on genome quality. Please give the assessment of genome assemblies using Merqury.

*Response: Quast results can be found here:*

*https://birneylab.github.io/MIKK_genome_companion_paper/Individual_assemblies/data/quast/report.html*

*We have also provided both BUSCO and Merqury assessment and added a short description of these results in the results and methods sections. The full results can be found here:*

*https://birneylab.github.io/MIKK_genome_companion_paper/Individual_assemblies/*

8.In Fig. 2A, "the possible reference deletion" should mean that the segment is deleted in reference HDRR, why it still belongs to reference (colored with HDRR)? Please make the illustration clear.

*Response: We have reworded the reference to this figure “whereas the connection of two non adjacent HdrR segments in linear space is indicative of possible deletions” which describes links between segments rather than segments themselves.*

9.The labels in x axis of Fig. 2F is incomplete.

*Response: We have corrected this.*

10.In page 12 line 15, the result of CNVs is absent.

*Response: We have removed reference to SV detected using Illumina alone and focus more on the comparison between linear and graph based approaches using the same data type (ONT polished with Illumina).*

11.In page 14 line 20-21, what about the function of these four "interesting" genes? Moreover, whether their expressions are changed owing to DMR?

*Response: We have added a short description of the four mentioned genes and have also investigated whether the predicted levels of methylation at these significant and closeby DMRs had an observable effect on expression (Additional File 3 Figure 4). The results here are limited due both to the relatively low number of available samples and limitations of bulk RNA sequence; however we do observe a significant (p=0.002) relationship between methylation likelihood and expression for the galm gene in the liver.*

12.In page 12 line 12 "similar to many human studies", please add reference.

*Response: Reference to a recent study into SV in the human genome added.*

**Second round of review**

**Reviewer 1**

The authors have addressed my previous comments in a satisfactory manner.

**Reviewer 2**

Actually, authors provided targeted answers based on the questions in this revision and the manuscript was improved. However, it’s a pity that there are still some issues need to be further elaborated.

1. It was mentioned that the presented here is for downstream analysis of pangenome reference graphs except sections of “the construction of pan genome graphs”, “De novo assembly of MIKK panel genomes“, “Variation pangenome graph assembly” and “Graph alignment and segment usage analysis”. However, what is the novelty or advance of the “methodology” compared with other publicly available software or pipelines? This information should be presented at least in the background and the discussion sections.

2. To our knowledge, a gene was considered core if it was present in all lines and variable if it was absent in at least one line (Golicz et al., 2016). The result you mentioned “We assessed the graph segment usage of MIKK panel lines in relation to HdrR gene content and found that of the 24,328 annotated genes, 92% are covered by graph segments used by at least one MIKK line. For the coding exons found within these genes 87% of coordinates are fully encompassed by a single graph segment, 13% partially covered or present across multiple segments with only 114 exons (0.03%) being fully absent in all MIKK panel lines.” was not the core genes of the pangenome. Please add the analysis in the result and method sections.

3. “For the BUSCO results using 3640 BUSCOs from 26 genomes most MIKK panel assemblies had greater than 50% complete BUSCOs with a median of 59% across the 12 assemblies. For the reference free assessment using Merqury we observe consistent consensus quality (QV) estimates and reasonable k-mer completeness values with a median completeness of 78% across all 12 assemblies”. The assessment value of BUSCO and Merqury were extremely inferior to our general knowledge (eg. Carassius auratus: 95.6%). It is not convinced of the genome assembly as the high quality of genome assembly is essential for the downstream analysis. Besides, please add the information of genome assessment in Table2 or supplementary files directly.

4. page14 line10-21, the comparation of the graph-based and reference-anchored approach, I can not find the provenience of “19 regions”、”16 regions”…that you mentioned in the context? Or is it sampling for comparation? Please point out the data or regions you used to compare clearly.

**Authors Response**

**Point-by-point responses to the reviewers’ comments:**

Reviewer #2:

Actually, authors provided targeted answers based on the questions in this revision and the manuscript was improved. However, it’s a pity that there are still some issues need to be further elaborated.

1. It was mentioned that the presented here is for downstream analysis of pangenome reference graphs except sections of “the construction of pan genome graphs”, “De novo assembly of MIKK panel genomes“, “Variation pangenome graph assembly” and “Graph alignment and segment usage analysis”. However, what is the novelty or advance of the “methodology” compared with other publicly available software or pipelines? This information should be presented at least in the background and the discussion sections.

*Response: We appreciate that we may not have adequately put this work in context or fully highlighted its novelty in terms of methodology. However, we have provided extensive details on how we have approached the use of graph genomes applied to the MIKK panel. By using graph representations, we were able to obtain a deeper characterisation of genetic variation present in MIKK panel lines and relative to 3 high quality medaka fish reference genomes. We have provided several examples of variations that 1) would be missed using the linear reference approach and 2) are likely to have a functional consequence.*

*We have added additional content to both the introduction and discussion highlighting that we provide a lot of custom methods and code for traversing graph genomes, detecting variation, and interpreting those variants. Please note that on review of these sections we already found multiple statements relating to the overall novelty of this work but have added new content relevant to the methodology developed during this work as well as putting the research into the context of the wider medaka research community. We have also rephrased parts of both the introduction and discussion to improve the description of its overall novelty and have added content specifically for methodological novelty at pages 5 and 6 (introduction) and 21 (discussion).*

2. To our knowledge, a gene was considered core if it was present in all lines and variable if it was absent in at least one line (Golicz et al., 2016). The result you mentioned “We assessed the graph segment usage of MIKK panel lines in relation to HdrR gene content and found that of the 24,328 annotated genes, 92% are covered by graph segments used by at least one MIKK line. For the coding exons found within these genes 87% of coordinates are fully encompassed by a single graph segment, 13% partially covered or present across multiple segments with only 114 exons (0.03%) being fully absent in all MIKK panel lines.” was not the core genes of the pangenome. Please add the analysis in the result and method sections.

*Response: We thank the reviewer for their patience on this item and for the reference provided - we were unfamiliar with this type of core gene analysis. We have repeated the analysis using the definition of core vs variable genes from (Golicz et al., 2016) and referenced accordingly. There is an interesting aspect here about how one defines a gene as “present” in all lines – however we have taken the same approach as (Golicz et al., 2016) and obtained some insightful results.*

*We have updated the description section at page 10 (Pangenome graph assembly and read alignment section and Supplementary Fig S2).*

3. “For the BUSCO results using 3640 BUSCOs from 26 genomes most MIKK panel assemblies had greater than 50% complete BUSCOs with a median of 59% across the 12 assemblies. For the reference free assessment using Merqury we observe consistent consensus quality (QV) estimates and reasonable k-mer completeness values with a median completeness of 78% across all 12 assemblies”. The assessment value of BUSCO and Merqury were extremely inferior to our general knowledge (eg. Carassius auratus: 95.6%). It is not convinced of the genome assembly as the high quality of genome assembly is essential for the downstream analysis. Besides, please add the information of genome assessment in Table2 or supplementary files directly.

*Response: Although the level of missing BUSCOs in our draft MIKK panel assemblies is higher than needed for reference grade assemblies, we observe similar levels compared to similar draft assemblies from other teleost species – see table 5 from (https://www.nature.com/articles/sdata2016132/). Importantly the closest available BUSCO lineage dataset is actinopterygii consisting of 3,698 highly conserved genes. Teleosts make up by far the largest infraclass of the large actinopterygii class (ray finned fish making up over 50% of living vertebrates) with over 26,000 different Teleost species having been described.*

*We acknowledge that our draft assemblies are less high quality based on BUSCO assessment then assemblies from other certain species however we obtain similar results to previous Teleost draft assemblies (https://www.nature.com/articles/sdata2016132/tables/6). The BUSCO and Merqury QC measures have been provided as a new supplementary table (Additional file 3: Table S2) as requested and we have updated the section describing these results at pages 7 and 8 (Individual MIKK line assemblies section).*

4. page14 line10-21, the comparation of the graph-based and reference-anchored approach, I can not find the provenience of “19 regions”、”16 regions”…that you mentioned in the context? Or is it sampling for comparation? Please point out the data or regions you used to compare clearly.

*Response: We have made this section clearer and added a reference to the relevant supplementary tables that set out the 19 and 16 regions respectively (Additional file 6: Table S5 and Additional file 7: Table S6).*
